# Supplementary figures and images for: ELF5 modulates the estrogen receptor cistrome in breast cancer
Source: PLoS Genet. 2020 Jan 2;16(1):e1008531. doi: 10.1371/journal.pgen.1008531 (PMC6959601; doi:10.1371/journal.pgen.1008531)

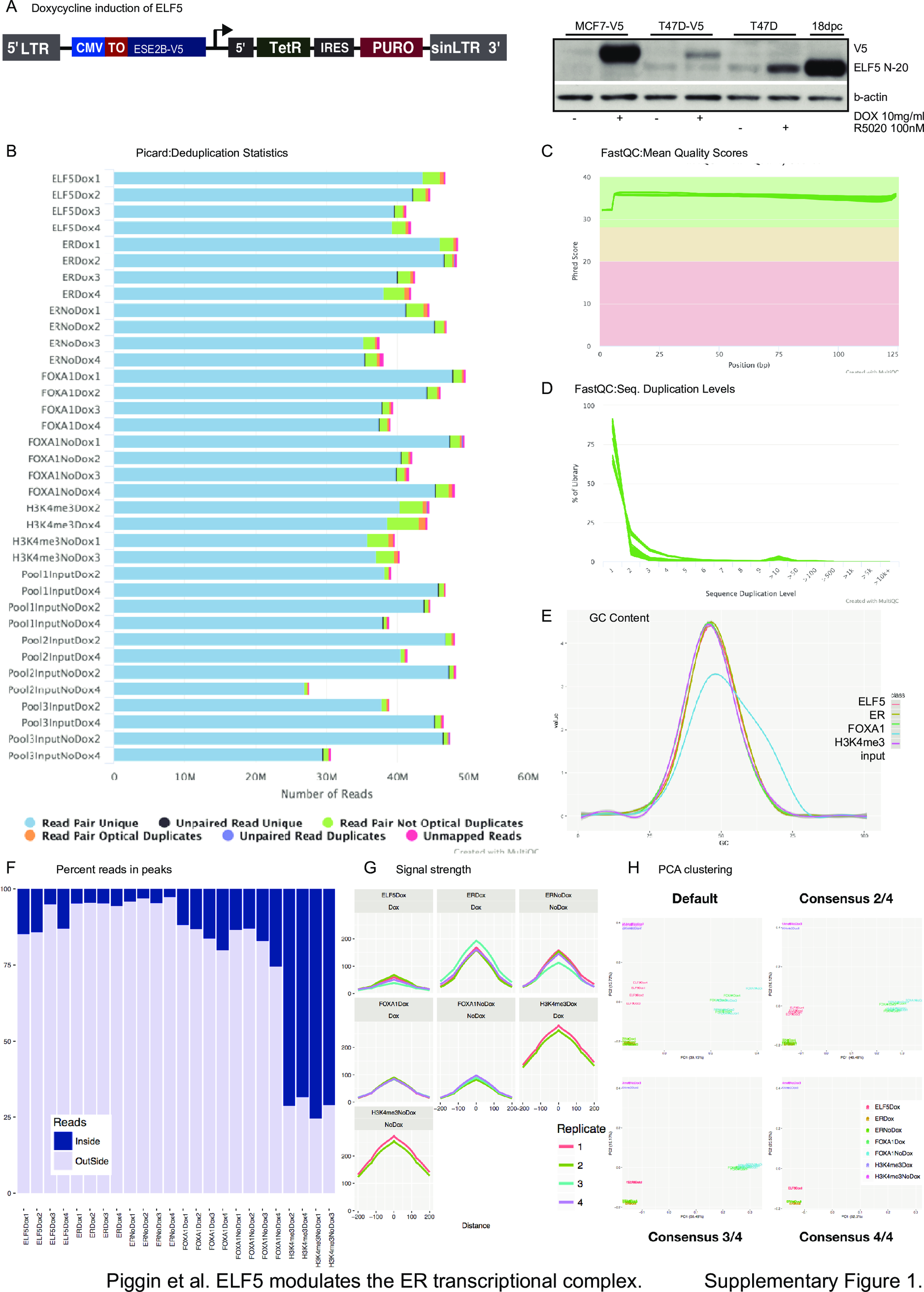

Supplement: S1 Fig — MCF-7 cells stably infected with either an empty retroviral doxycycline-inducible vector, or one expressing isoform 2 of human ELF5 tagged with V5 [19] (Panel A), were treated with 0.1ug/ml doxycycline for 48 hours prior to cross-linking and processing for ChIP-seq using antibodies precipitating ELF5, ER, FOXA1 or H4K4me3 together with inputs. Four independent replicates for each ChIP (2 for H3K4me3) were conducted. A set of standard quality control measures were evaluated. Panel A, ELF5 expression level before and after induction with DOX compared to levels of induction achieved in T-47D cells with R5020 [19], or in mammary gland by 18 days of pregnancy in mice. Panel B, Read depth and errors, panel C, paired read score, panel D sequence duplication, panel E GC content, panel F, reads in peaks and panel G, signal strength. Panel H, principle components analysis (PCA), showed the presence of a peak in 3 of 4 replicates correctly grouped the ChIP replicates and separated all of the FOXA1 plus and minus dox replicates. (TIF) [file pgen.1008531.s001.tif]

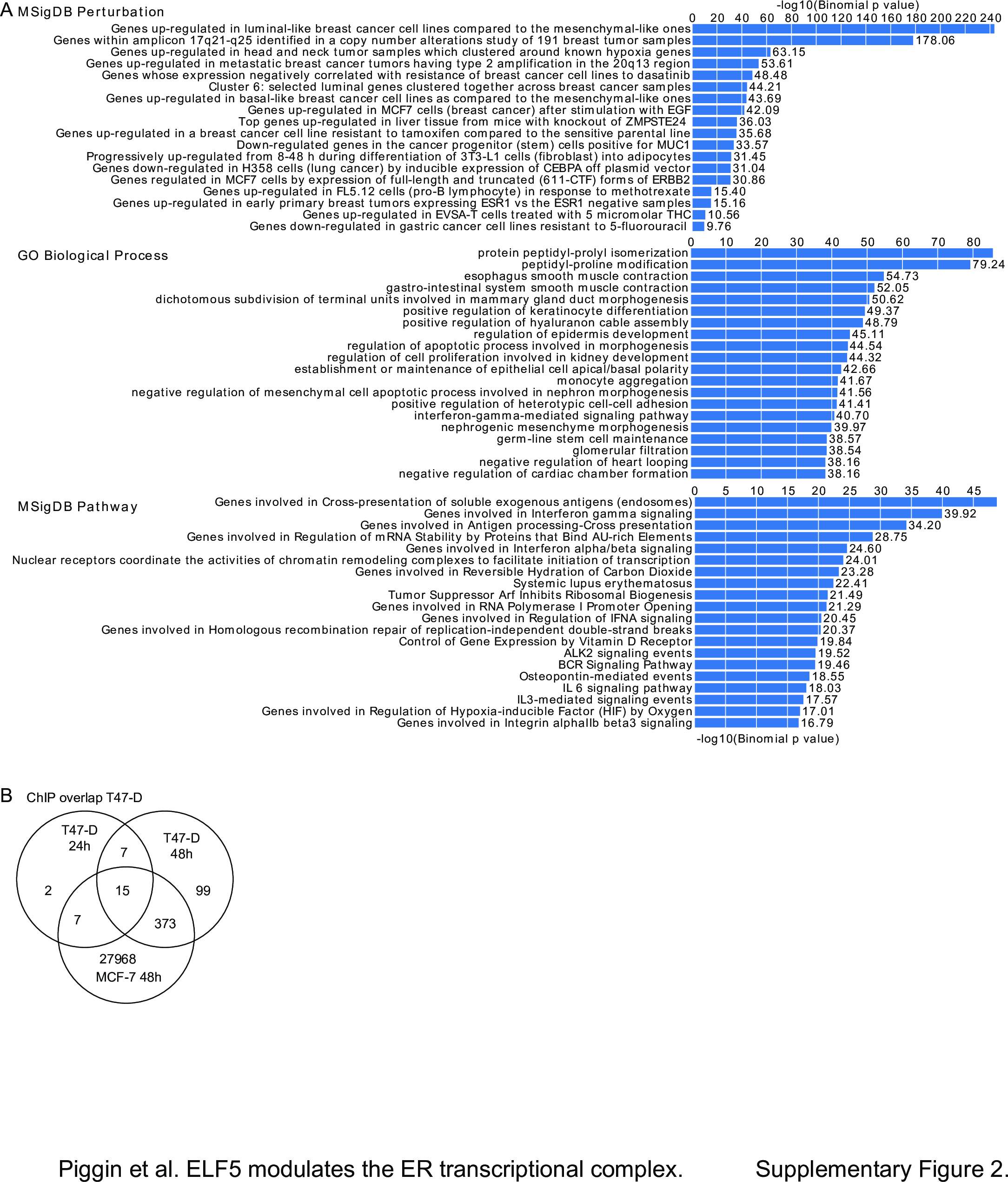

Supplement: S2 Fig — Panels A, GREAT functional analysis of ELF5 genomic binding using MSigDB gene sets as indicated. Panel B, overlap of MCF-7 ChIP peaks with those observed in T-47D cells [19]. (TIF) [file pgen.1008531.s002.tif]

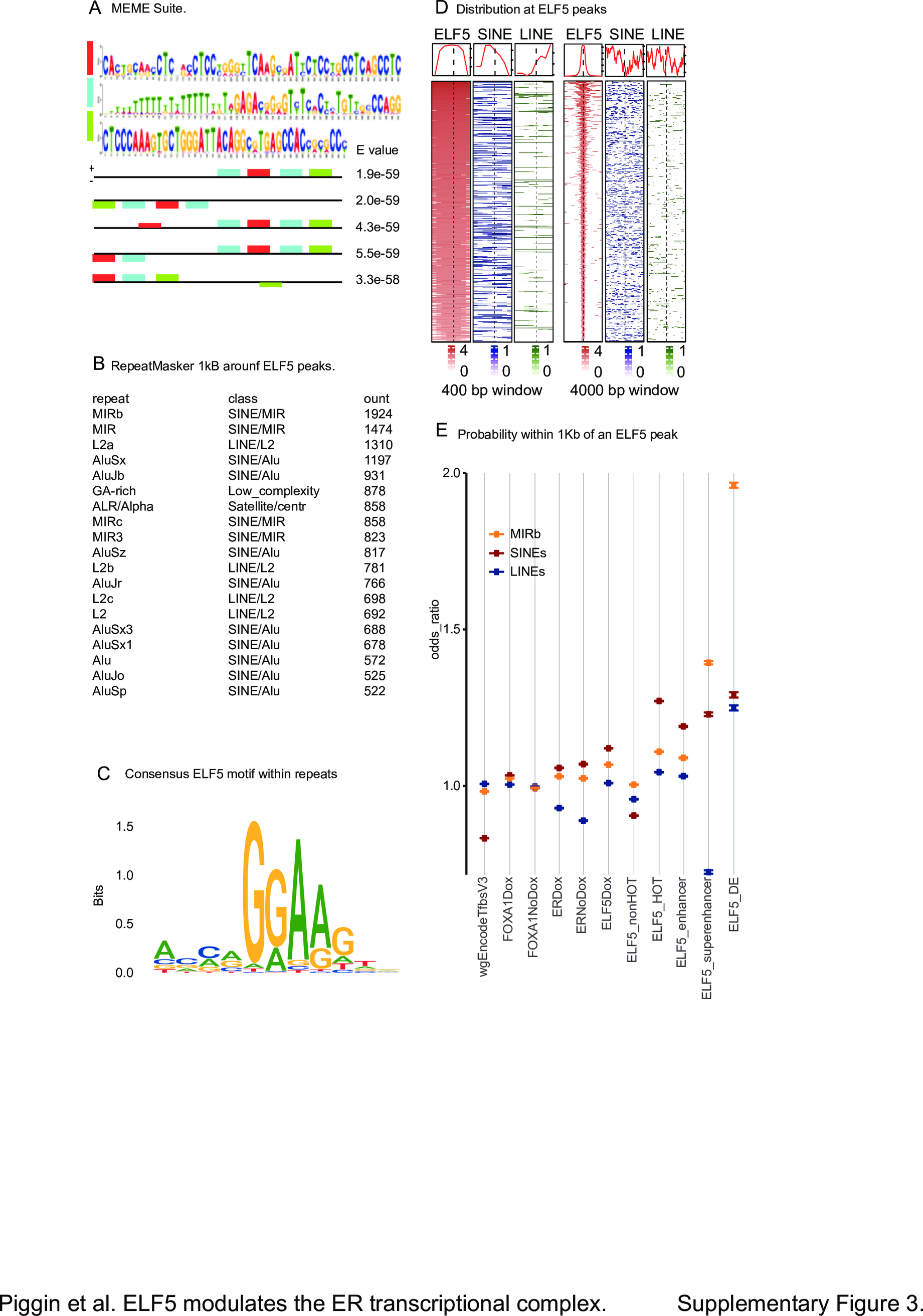

Supplement: S3 Fig — Panel A, sequence of motifs at ELF5 binding sites with identity to Alu repeats (DFAM) with red blue and green color bars showing the most frequent arrangements of these motifs and their enrichment (E) p value. Panel B, RepeatMasker analysis of repeat sequences at ELF5 binding sites showing number and type detected. Panel C, consensus ETS motif under ELF5 binding sites at repeats. Panel D, distribution of the indicated repeat types around ELF5 binding sites at the indicated window sizes. Panel E, odds ratios for finding the indicated repeat types under all transcription factor binding sites (wgEncode TfbsV3), under FoxA1, ER and ELF5 with or without DOX treatment, then at ELF5 binding sites within highly occupied target regions (HOT), enhancers (E), super enhancers (SE) or in the vicinity of differentially expressed genes (DE). Error bars represent standard error. (TIF) [file pgen.1008531.s003.tif]

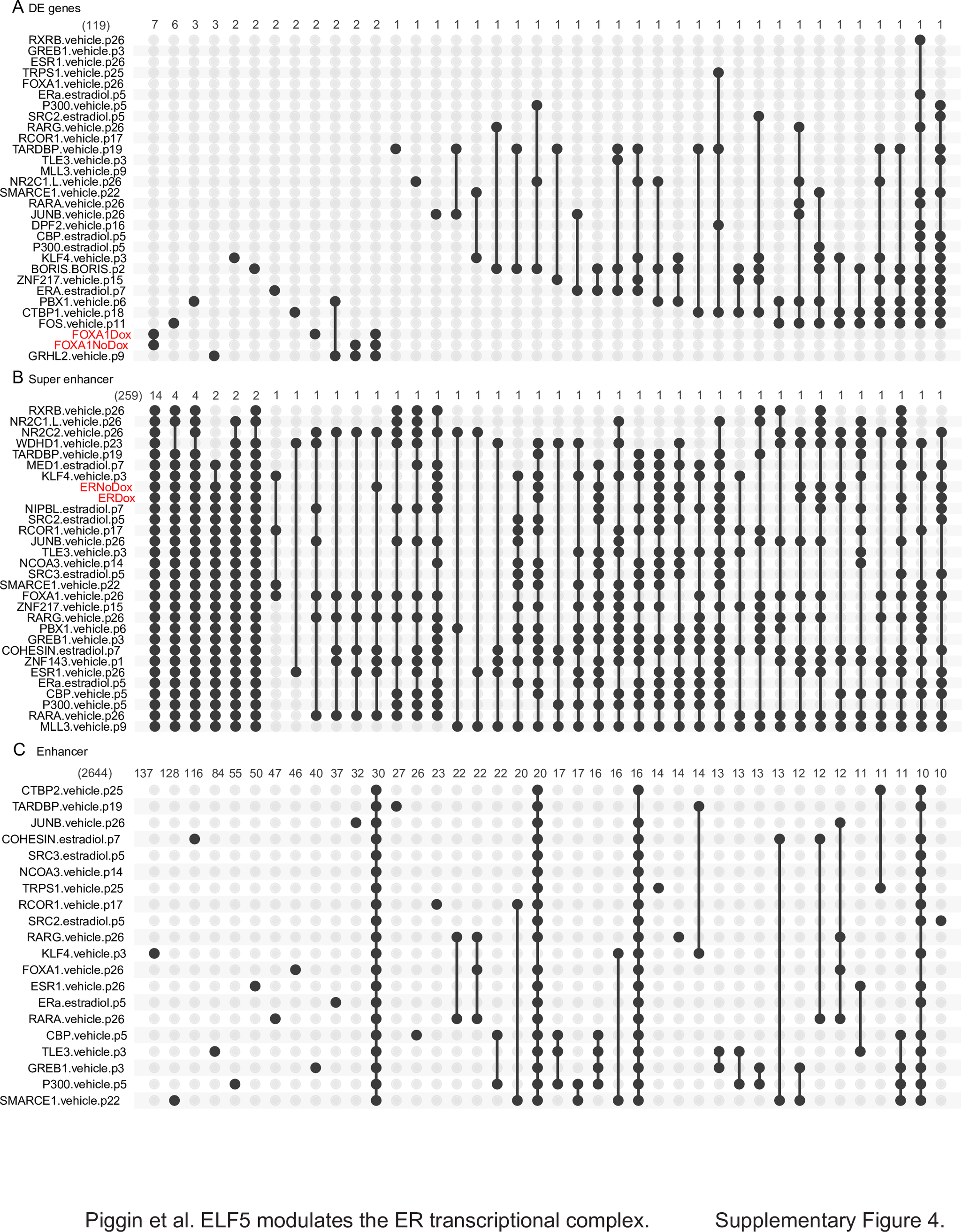

Supplement: S4 Fig — UpSet analysis, using the transcription factors whose binding is most frequently co-located with ELF5, to identify patterns of co-binding at differentially expressed (DE) genes, (Panel A 119 genomic loci), super enhancers (Panel B 259 loci) and enhancers (Panel C 2644 loci). Numbers above the transcription factor sets show the number instances of that specific set. Black dots indicate the presence within the set of the indicated transcription factor. (TIF) [file pgen.1008531.s004.tif]

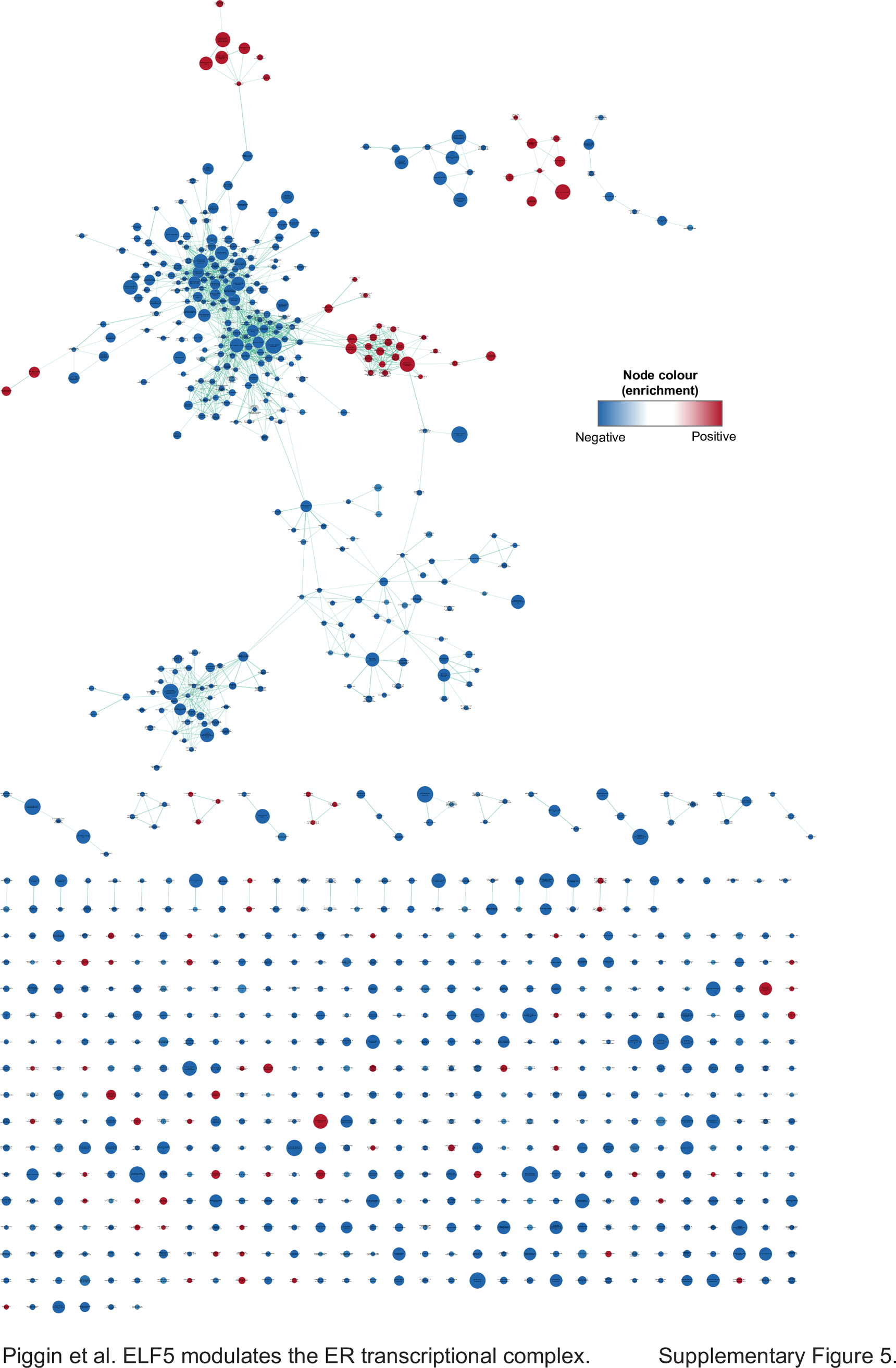

Supplement: S5 Fig — Scalable .pdf showing complete Cytoscape representation of the RNA-seq data. Each circle (node) is sized to indicate the relative number of genes in the set and coloured to show enrichment score in response to ELF5. Nodes with overlaps in their gene content are linked by green lines and are clustered according to the degree of overlap. Download and zoom to see the detail. (TIF) [file pgen.1008531.s005.tif]

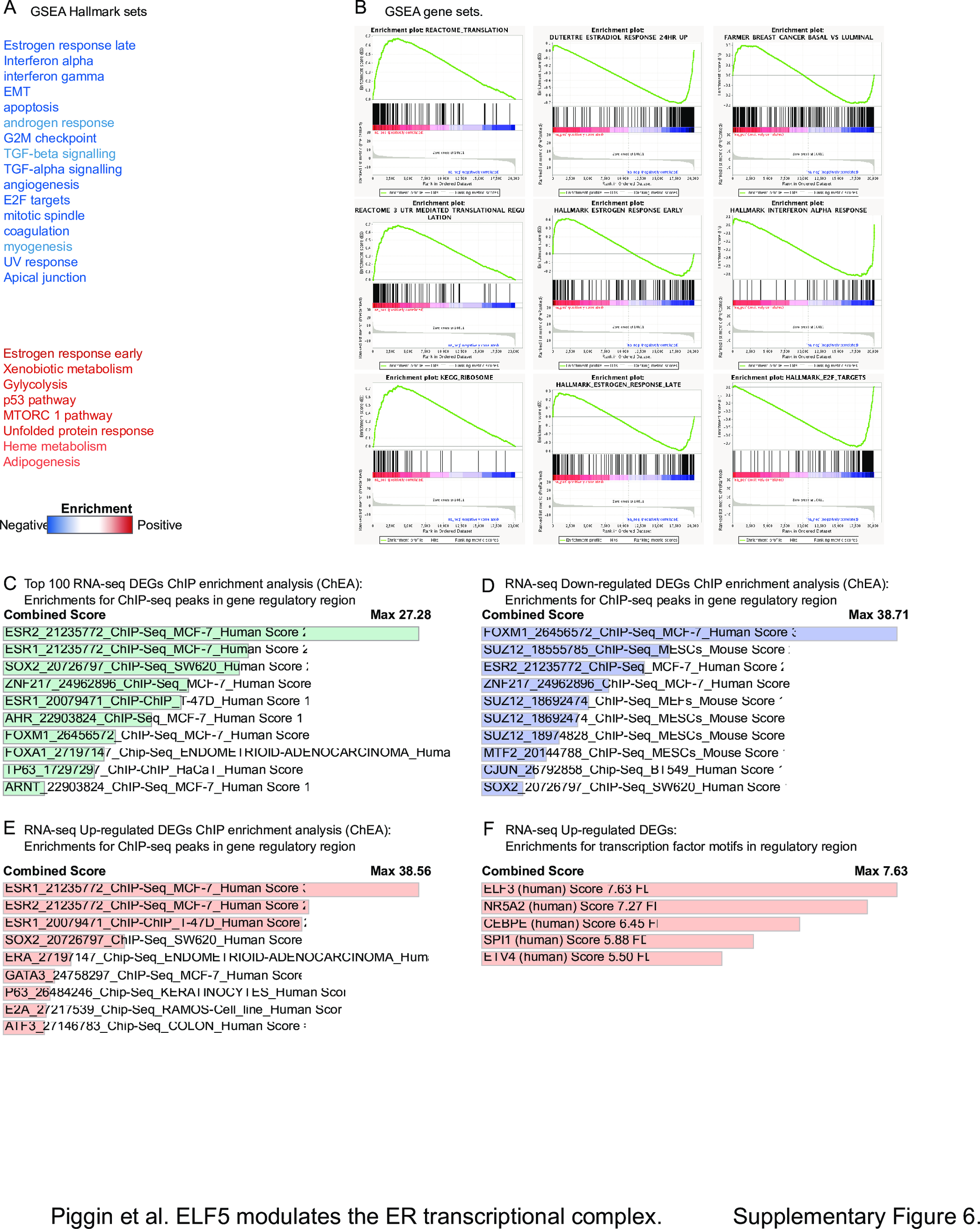

Supplement: S6 Fig — Panel A, GSEA of MSigDB Hallmark gene sets coloured according to enrichment score as indicated by the scale. Panel B, example GSEA plots from the MSigDB C2-all sets showing significant enrichment. Panel C, enriched ChIP sets (ranked by Enrichr combined score) identified in the regulatory regions of the top 100 differentially expressed MCF7-ELF5 RNA-seq genes (filtered for absolute fold-change >1.5 and ranked by FDR). The identifier for each ChIP set contains the name of the transcription factor followed by the PubMed ID, the type of experiment (ChIP-seq or ChIP-chip), the cell line or tissue, and the species. The top 10 sets (of 37 sets with an FDR <0.05) are shown. Analysis was performed using the Enrichr ChIP enrichment analysis (ChEA) tool. Panel D, enriched ChIP sets identified in the regulatory regions of down-regulated genes. Panel E, enriched ChIP sets identified in the regulatory regions of up-regulated genes. Panel F, enriched transcription factor motifs in ELF5 regulated genes from the TRANSFAC and JASAPR databases. No enriched motifs were identified for the down-regulated RNA-seq genes. (TIF) [file pgen.1008531.s006.tif]

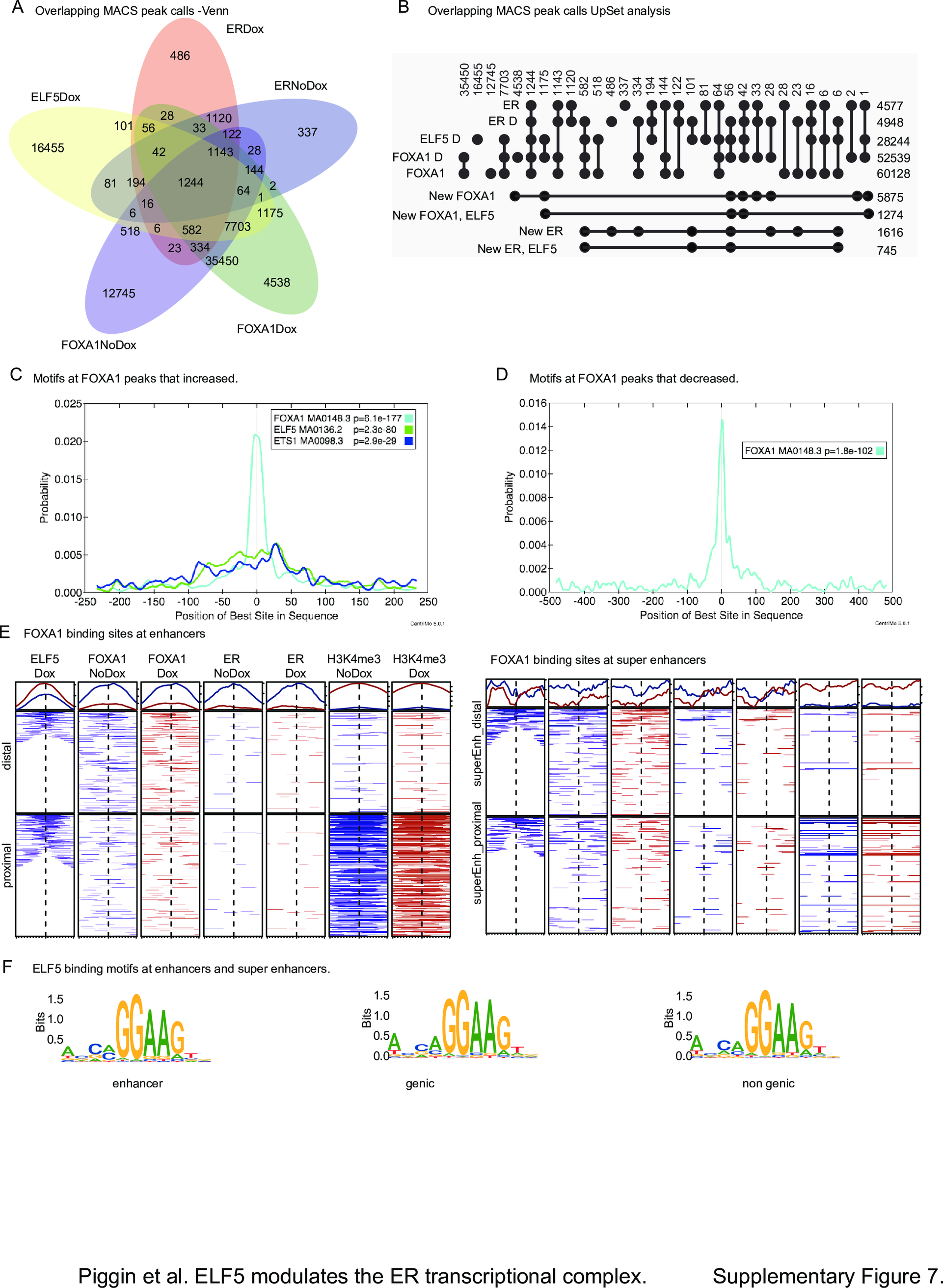

Supplement: S7 Fig — Panel A. Venn diagram for co-occurrence of binding peaks called present by MACS for ELF5, FOXA1 and ER. Panel B, matching UpSet plot for co-occurrence of binding peaks called present by MACS. Panel C, motif probability analysis of sequences under FOXA1 binding sites that were increased by induction of ELF5. Panel D), motif probability analysis of sequences under FOXA1 binding sites that were decreased by induction of ELF5. Panel E, ELF5, FOXA1, ER and H3K4me3 peaks are plotted, before and after induction of ELF5, centred on all FOXA1 summits that overlap enhancers and superenhancers. Color scale denotes log 10 of MACS score. Panel F, consensus ETS motifs under ELF5 binding sites in the indicated genomic regions. (TIF) [file pgen.1008531.s007.tif]

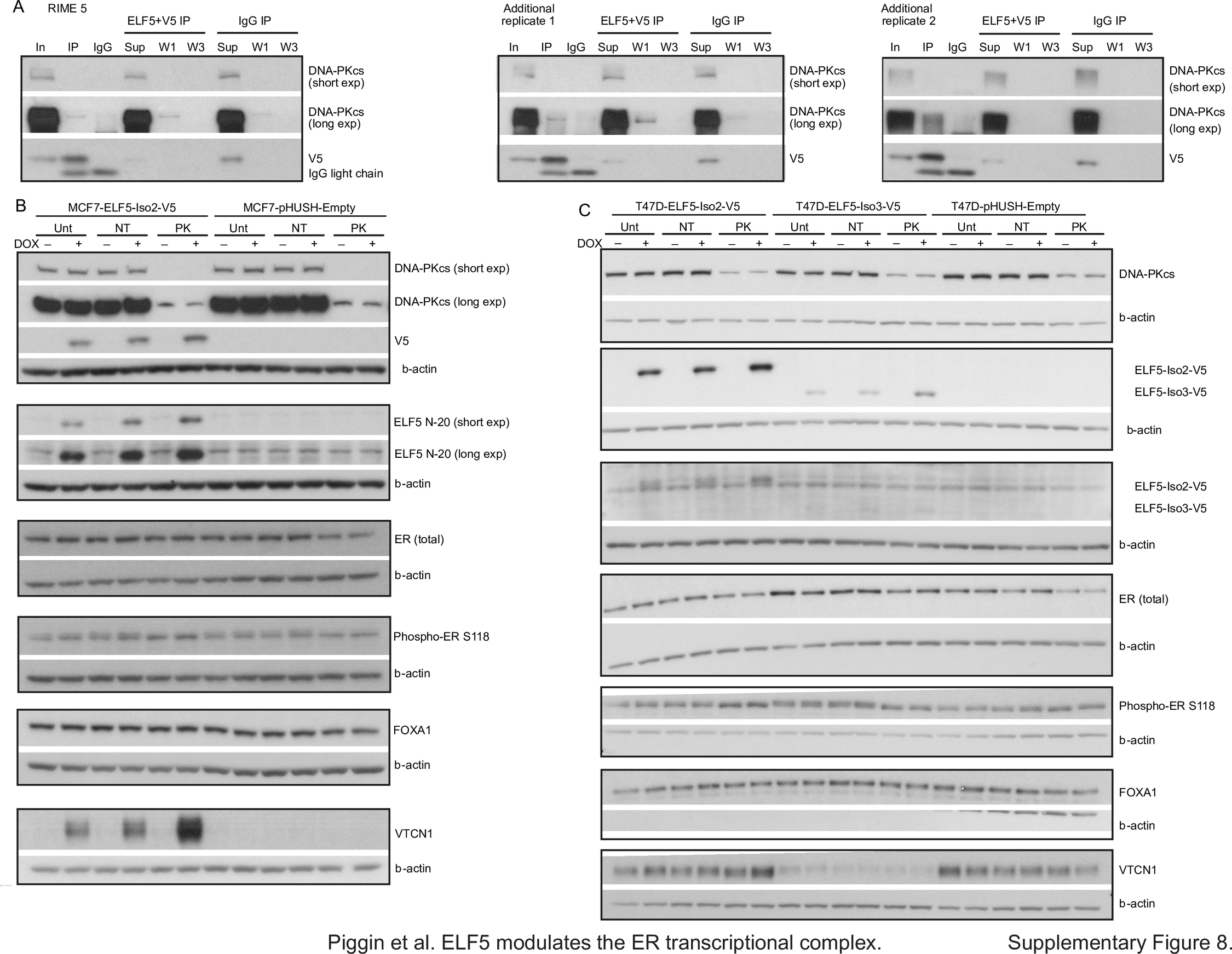

Supplement: S8 Fig — Panel A, immunoprecipitation of ELF5 co-precipitates DNA-PKcs. Samples were prepared using the RIME protocol and immunoprecipitated with a combination of ELF5 and V5 antibodies or IgG control. Blots for V5 and DNA-PKcs are shown. Lane 1 is the input or total lysate (In), lane 2 is the ELF5-V5 immunoprecipitation (IP) and lane 3 is the IgG control immunoprecipitation (IgG). Lanes 4 and 7 are supernatants from the immunoprecipitations (Sup, representing unbound protein), while lanes 5–6 and 8–9 are supernatants from the first (W1) and third (W3) bead washes (indicating no residual unbound protein after the final third wash). RIME 5 is replicate 5 of the ELF5-V5 RIME experiments, while additional replicates 1 and 2 did not form part of the ELF5-V5 RIME dataset. Panel B and C, Western blots for MCF-7 (panel B) and T-47D (panel C) cell lines, stably modified with doxycycline-inducible pHUSH-ELF5 isoform 2 or isoform 3 vector (empty vector as a control). Cells were untransfected (Unt), transfected with a non-targeting siRNA (NT) or transfected with siRNA targeting DNA-PKcs (PK). Cells were also treated with doxycycline (Dox, indicated by + symbol) or vehicle (-). Each box represents an individual blot and is shown with the corresponding beta-actin (b-actin) loading control. (TIF) [file pgen.1008531.s008.tif]

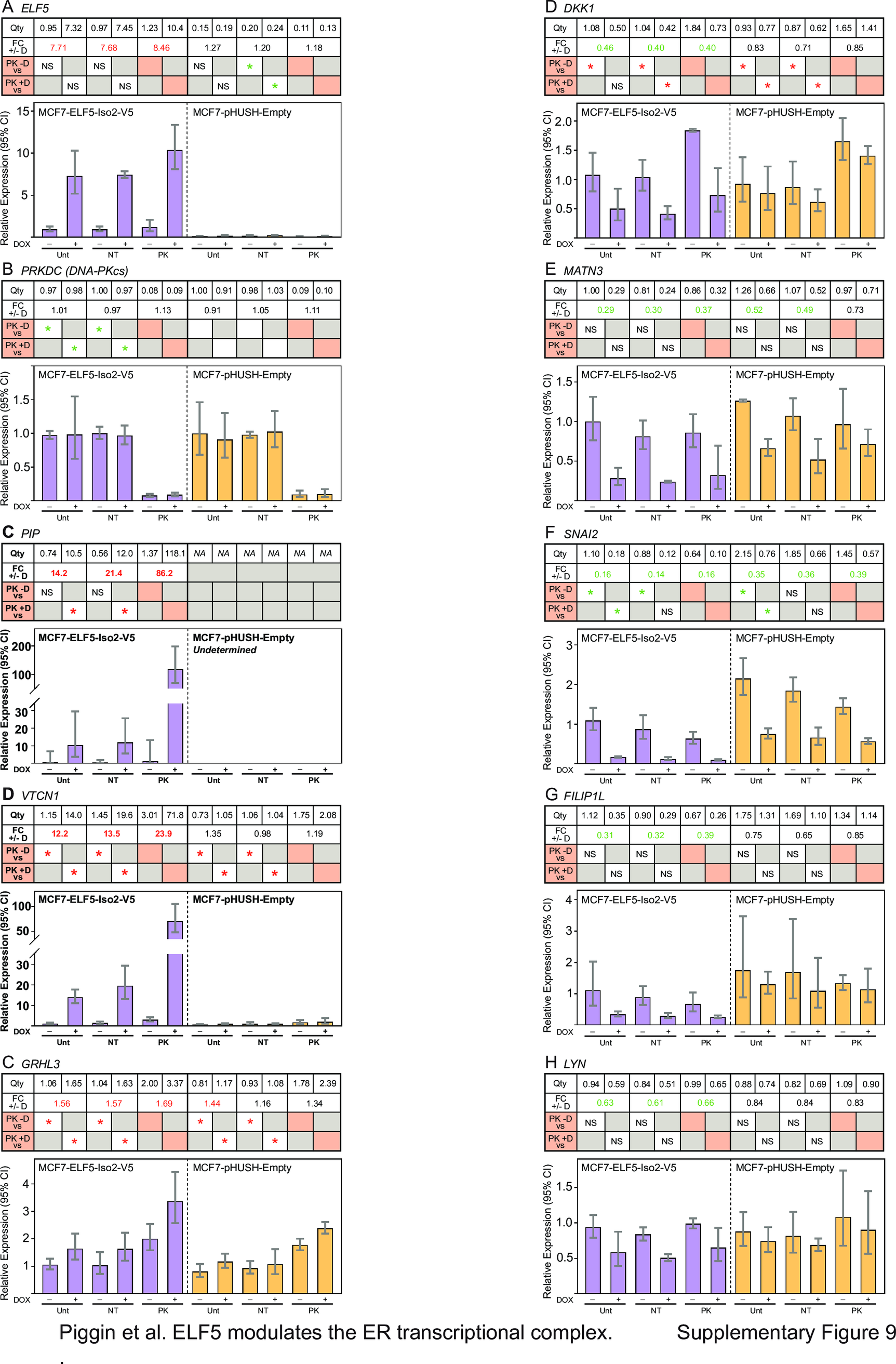

Supplement: S9 Fig — Panels A-H, Effect of DNA-PKcs knockdown on ELF5-driven changes in expression of the indicated genes in MCF-7 and T47-D cells. Fold change is indicated in red where DNA-PKcs exerted a significant suppression of gene expression and in green where DNA-PKcs enhanced expression. Cells were untransfected (Unt), transfected with a non-targeting siRNA (NT) or transfected with siRNA targeting DNA-PKcs (PK). Cells were also treated with doxycycline (Dox, +) or vehicle (-). Graphs show the mean calibrated normalised relative quantity values from three biological replicates with 95% confidence interval. The associated table, vertically aligned with the corresponding samples in the graph, provides the exact mean normalised quantity value (Qty, row 1). Row 2 of the table indicates the effects of ELF5 induction on the target gene expression; the fold changes for the vertically aligned +Dox and -Dox sample pairs are shown, with red typeface indicating a significant upregulation (one-way ANOVA), green a significant downregulation and black a non-significant fold change. Rows 3 and 4 of the table indicate the effect of DNA-PKcs knockdown on the target gene expression. Row 3 compares the siPK -Dox sample (indicated by the orange box) with each of the Unt -Dox and the siNT -Dox samples, with a red asterisk indicating a significant upregulation and a green asterisk a significant downregulation (NS = no significant difference, one-way ANOVA). Similarly, row 4 compares the siPK +Dox sample (indicated by the orange box) with each of the Unt +Dox and siNT +Dox samples. (TIF) [file pgen.1008531.s009.tif]
