# Supplementary material for: ELF5 modulates the estrogen receptor cistrome in breast cancer
Source: PLoS Genet. 2020 Jan 2;16(1):e1008531. doi: 10.1371/journal.pgen.1008531 (PMC6959601; doi:10.1371/journal.pgen.1008531)
Supplement: S1 Table — Proteins are listed by the level of stringency required to include them, by progressive relaxing the requirements for presence in the replicates, as indicated. (PDF) [file pgen.1008531.s010.pdf]

## Supplementary Table 1: ELF5-interacting proteins identified by RIME

| Uniprot ID | Uniprot Protein Name | Unique Peptides | | | | | Mascot Score | | | | |
|---|---|---|---|---|---|---|---|---|---|---|---|
| | | 1 (%) | 2 | 3 | 4 | 5 | 1 | 2 | 3 | 4 | 5 |
| **5 of 5 replicates (3)** | | | | | | | | | | | |
| ELF5_HUMAN | ETS-related transcription factor Elf-5 | 4 (15.5) | 4 | 2 | 4 | 5 | 490 | 587 | 146 | 213 | 365 |
| PRKDC_HUMAN | DNA-dependent protein kinase catalytic subunit | 15 (4.8) | 6 | 1 | 1 | 1 | 239 | 128 | 42 | 47 | 51 |
| SC16A_HUMAN | Protein transport protein Sec16A | 14 (12.5) | 20 | 7 | 1 | 2 | 159 | 1244 | 278 | 85 | 202 |
| **4 of 5 replicates (5)** | | | | | | | | | | | |
| RL13A_HUMAN | 60S ribosomal protein L13a | 4 (22.7) | 1 | 2 | | 1 | 80 | 51 | 118 | | 73 |
| RL18A_HUMAN | 60S ribosomal protein L18a | | 2 | 2 | 1 | 1 | | 85 | 123 | 57 | 54 |
| RL30_HUMAN | 60S ribosomal protein L30 | 2 (17.4) | 2 | 2 | | 1 | 29 | 121 | 78 | | 84 |
| TRI25_HUMAN | E3 ubiquitin/ISG15 ligase TRIM25 | 2 (3.5) | 1 | | 1 | 1 | 148 | 43 | | 51 | 55 |
| U2AF1_HUMAN | Splicing factor U2AF 35 kDa subunit | 2 (10.8) | 2 | 1 | | 1 | 56 | 111 | 68 | | 62 |
| **3 of 5 replicates (13)** | | | | | | | | | | | |
| ATPA_HUMAN | ATP synthase subunit alpha, mitochondrial | 1 (4.0) | 1 | | | 1 | 35 | 69 | | | 90 |
| H12_HUMAN | Histone H1.2 | 7 (17.8) | 2 | 1 | | | 479 | 126 | 98 | | |
| H2B1C_HUMAN | Histone H2B type 1-C/E/F/G/I | | | 2 | 3 | 2 | | | 75 | 96 | 89 |
| PAIRB_HUMAN | Plasminogen activator inhibitor 1 RNA-binding protein | 2 (7.8) | 1 | | | 1 | 62 | 81 | | | 84 |
| PPM1G_HUMAN | Protein phosphatase 1G | 8 (25.5) | 3 | 1 | | | 186 | 100 | 48 | | |
| PUR6_HUMAN | Multifunctional protein ADE2 | 2 (10.4) | 1 | | | 1 | 87 | 62 | | | 109 |
| RA1L2_HUMAN | Heterogeneous nuclear ribonucleoprotein A1-like 2 | | 2 | 1 | | 2 | | 160 | 48 | | 134 |
| RCC1_HUMAN | Regulator of chromosome condensation | 4 (15.7) | 1 | | | 1 | 27 | 129 | | | 45 |
| RS27A_HUMAN | Ubiquitin-40S ribosomal protein S27a | 3 (27.6) | 4 | 1 | | | 84 | 276 | 154 | | |
| TCPE_HUMAN | T-complex protein 1 subunit epsilon | 1 (4.3) | 1 | | | 1 | 61 | 87 | | | 53 |
| TRI33_HUMAN | E3 ubiquitin-protein ligase TRIM33 | 3 (2.9) | 3 | 1 | | | 138 | 102 | 64 | | |
| U2AF2_HUMAN | Splicing factor U2AF 65 kDa subunit | 1 (2.1) | 1 | | | 1 | 28 | 43 | | | 98 |
| XRCC5_HUMAN | X-ray repair cross-complementing protein 5 | 13 (30.1) | 2 | 1 | | | 166 | 50 | 45 | | |
| **2 of 5 replicates (53)** | | | | | | | | | | | |
| ACL6A_HUMAN | Actin-like protein 6A | 1 (4.4) | 1 | | | | 21 | 38 | | | |
| ACTG_HUMAN | Actin, cytoplasmic 2 | | 6 | 9 | | | | 744 | 465 | | |
| ATPB_HUMAN | ATP synthase subunit beta, mitochondrial | | 1 | 2 | | | | 77 | 82 | | |
| C2TA_HUMAN | MHC class II transactivator | | 1 | 1 | | | | 38 | 42 | | |
| CBX1_HUMAN | Chromobox protein homolog 1 | 1 (17.8) | 1 | | | | 181 | 130 | | | |
| CCAR2_HUMAN | Cell cycle and apoptosis regulator protein 2 | 11 (21.0) | 1 | | | | 358 | 55 | | | |
| CHD4_HUMAN | Chromodomain-helicase-DNA-binding protein 4 | 1 (0.9) | 1 | | | | 27 | 62 | | | |
| CLIC1_HUMAN | Chloride intracellular channel protein 1 | 1 (7.5) | 2 | | | | 48 | 116 | | | |
| CO3A1_HUMAN | Collagen alpha-1(III) chain | 2 (5.2) | 3 | | | | 91 | 117 | | | |
| COPB_HUMAN | Coatomer subunit beta | 2 (5.1) | 1 | | | | 24 | 83 | | | |
| DDX46_HUMAN | Probable ATP-dependent RNA helicase DDX46 | 1 (1.9) | 1 | | | | 20 | 45 | | | |
| DUS23_HUMAN | Dual specificity protein phosphatase 23 | 5 (37.3) | 1 | | | | 195 | 46 | | | |
| DX39A_HUMAN | ATP-dependent RNA helicase DDX39A | 2 (17.8) | 1 | | | | 171 | 55 | | | |
| H10_HUMAN | Histone H1.0 | 1 (4.6) | 1 | | | | 23 | 58 | | | |
| HNRC1_HUMAN | Heterogeneous nuclear ribonucleoprotein C-like 1 | | | | 1 | 1 | | | | 91 | 87 |

| Uniprot ID | Uniprot Protein Name | Unique Peptides | | | | | Mascot Score | | | | |
|---|---|---|---|---|---|---|---|---|---|---|---|
| | | 1 (%) | 2 | 3 | 4 | 5 | 1 | 2 | 3 | 4 | 5 |
| IF2A_HUMAN | Eukaryotic translation initiation factor 2 subunit 1 | 3 (11.8) | | 1 | | | 87 | | 66 | | |
| IMA6_HUMAN | Importin subunit alpha-6 | 1 (2.6) | 1 | | | | 41 | 37 | | | |
| LAP2A_HUMAN | Lamina-associated polypeptide 2, isoform alpha | 4 (15.6) | 1 | | | | 178 | 59 | | | |
| LASP1_HUMAN | LIM and SH3 domain protein 1 | 3 (21.8) | 2 | | | | 33 | 72 | | | |
| LC7L2_HUMAN | Putative RNA-binding protein Luc7-like 2 | 2 (5.9) | | | | 1 | 38 | | | | 63 |
| MCM3_HUMAN | DNA replication licensing factor MCM3 | 2 (4.3) | 2 | | | | 60 | 115 | | | |
| MCM5_HUMAN | DNA replication licensing factor MCM5 | 2 (3.4) | 1 | | | | 24 | 96 | | | |
| MCM6_HUMAN | DNA replication licensing factor MCM6 | 1 (1.6) | 1 | | | | 28 | 48 | | | |
| NOG1_HUMAN | Nucleolar GTP-binding protein 1 | 1 (2.7) | 1 | | | | 44 | 41 | | | |
| NP1L1_HUMAN | Nucleosome assembly protein 1-like 1 | 2 (7.4) | 1 | | | | 65 | 89 | | | |
| NPM3_HUMAN | Nucleoplasmin-3 | | 1 | 1 | | | | 133 | 71 | | |
| PRP19_HUMAN | Pre-mRNA-processing factor 19 | 3 (18.9) | 1 | | | | 114 | 44 | | | |
| PSA4_HUMAN | Proteasome subunit alpha type-4 | 1 (3.8) | 1 | | | | 64 | 69 | | | |
| PTBP2_HUMAN | Polypyrimidine tract-binding protein 2 | 16 (62.3) | 4 | | | | 1059 | 250 | | | |
| RAVR1_HUMAN | Ribonucleoprotein PTB-binding 1 | 17 (43.9) | 5 | | | | 1478 | 260 | | | |
| RAVR2_HUMAN | Ribonucleoprotein PTB-binding 2 | 12 (39.4) | 6 | | | | 505 | 271 | | | |
| RL17_HUMAN | 60S ribosomal protein L17 | 3 (15.8) | | 2 | | | 72 | | 61 | | |
| RL35A_HUMAN | 60S ribosomal protein L35a | 2 (22.7) | | 1 | | | 49 | | 43 | | |
| RL5_HUMAN | 60S ribosomal protein L5 | 4 (16.2) | 1 | | | | 67 | 53 | | | |
| RMXL1_HUMAN | RNA binding motif protein, X-linked-like-1 | | 1 | 1 | | | | 50 | 61 | | |
| RS12_HUMAN | 40S ribosomal protein S12 | 2 (13.6) | 1 | | | | 34 | 98 | | | |
| RS27L_HUMAN | 40S ribosomal protein S27-like | 1 (28.6) | | 1 | | | 32 | | 55 | | |
| SC23A_HUMAN | Protein transport protein Sec23A | | 5 | 1 | | | | 257 | 41 | | |
| SC23B_HUMAN | Protein transport protein Sec23B | 2 (7.0) | 8 | | | | 26 | 419 | | | |
| SRSF9_HUMAN | Serine/arginine-rich splicing factor 9 | 3 (19.0) | 1 | | | | 34 | 50 | | | |
| SSRP1_HUMAN | FACT complex subunit SSRP1 | 3 (5.2) | 2 | | | | 53 | 122 | | | |
| SYEP_HUMAN | Bifunctional glutamate/proline--tRNA ligase | 2 (2.4) | | | | 1 | 27 | | | | 45 |
| SYFA_HUMAN | Phenylalanine--tRNA ligase alpha subunit | 1 (2.8) | 1 | | | | 45 | 83 | | | |
| TCP4_HUMAN | Activated RNA polymerase II transcriptional coactivator p15 | 2 (22.1) | 2 | | | | 30 | 98 | | | |
| TCPG_HUMAN | T-complex protein 1 subunit gamma | 6 (19.6) | 1 | | | | 133 | 62 | | | |
| TCPH_HUMAN | T-complex protein 1 subunit eta | 2 (5.7) | | | | 1 | 57 | | | | 56 |
| TCPQ_HUMAN | T-complex protein 1 subunit theta | 2 (6.6) | 1 | | | | 56 | 115 | | | |
| THOC4_HUMAN | THO complex subunit 4 | 4 (31.5) | 1 | | | | 97 | 56 | | | |
| TLE1_HUMAN | Transducin-like enhancer protein 1 | | 1 | 1 | | | | 62 | 42 | | |
| TOP2B_HUMAN | DNA topoisomerase 2-beta | 2 (3.8) | 1 | | | | 202 | 57 | | | |
| UBF1_HUMAN | Nucleolar transcription factor 1 | 2 (6.0) | 1 | | | | 24 | 53 | | | |
| VDAC2_HUMAN | Voltage-dependent anion-selective channel protein 2 | 2 (7.5) | | 1 | | | 66 | | 48 | | |
| XPO2_HUMAN | Exportin-2 | 1 (3.0) | 2 | | | | 54 | 153 | | | |
