# Supplementary material for: ELF5 modulates the estrogen receptor cistrome in breast cancer
Source: PLoS Genet. 2020 Jan 2;16(1):e1008531. doi: 10.1371/journal.pgen.1008531 (PMC6959601; doi:10.1371/journal.pgen.1008531)
Supplement: S2 Table — Comparison to proteins known to interact with ER in MCF-7 cells [34] compared to those identified to interact with ELF5 by RIME in MCF-7 cells. (PDF) [file pgen.1008531.s011.pdf]

Supplementary Table 2: Common proteins identified in ELF5-V5 and ER RIME (MCF7 cells)

Accession	Uniprot ID	Protein Name
P12814	ACTN1_HUMAN	Alpha-actinin-1
Q9BTT0	AN32E_HUMAN	Acidic leucine-rich nuclear phosphoprotein 32 family member E
P45973	CBX5_HUMAN	Chromobox protein homolog 5
Q8N163	CCAR2_HUMAN	Cell cycle and apoptosis regulator protein 2
Q14839	CHD4_HUMAN	Chromodomain-helicase-DNA-binding protein 4
Q9UNE7	CHIP_HUMAN	E3 ubiquitin-protein ligase CHIP
P56545	CTBP2_HUMAN	C-terminal-binding protein 2
Q6NXG1	ESRP1_HUMAN	Epithelial splicing regulatory protein 1
P23771	GATA3_HUMAN	Trans-acting T-cell-specific transcription factor GATA-3
Q6ISB3	GRHL2_HUMAN	Grainyhead-like protein 2 homolog
P78347	GTF2I_HUMAN	General transcription factor II-I
O14929	HAT1_HUMAN	Histone acetyltransferase type B catalytic subunit
Q92769	HDAC2_HUMAN	Histone deacetylase 2
Q9BUJ2	HNRL1_HUMAN	Heterogeneous nuclear ribonucleoprotein U-like protein 1
O60341	KDM1A_HUMAN	Lysine-specific histone demethylase 1A
P42166	LAP2A_HUMAN	Lamina-associated polypeptide 2, isoform alpha
P25205	MCM3_HUMAN	DNA replication licensing factor MCM3
P33991	MCM4_HUMAN	DNA replication licensing factor MCM4
P33992	MCM5_HUMAN	DNA replication licensing factor MCM5
P55209	NP1L1_HUMAN	Nucleosome assembly protein 1-like 1
Q9ULU4	PKCB1_HUMAN	Protein kinase C-binding protein 1
P30041	PRDX6_HUMAN	Peroxiredoxin-6
O95758	PTBP3_HUMAN	Polypyrimidine tract-binding protein 3
Q16576	RBBP7_HUMAN	Histone-binding protein RBBP7
Q9BWF3	RBM4_HUMAN	RNA-binding protein 4
Q92785	REQU_HUMAN	Zinc finger protein ubi-d4
P42677	RS27_HUMAN	40S ribosomal protein S27
Q15637	SF01_HUMAN	Splicing factor 1
O75533	SF3B1_HUMAN	Splicing factor 3B subunit 1
Q15427	SF3B4_HUMAN	Splicing factor 3B subunit 4
Q969G3	SMCE1_HUMAN	SWI/SNF-related matrix-associated actin-dependent regulator of chromatin E1
Q9BZK7	TBL1R_HUMAN	F-box-like/WD repeat-containing protein TBL1XR1
P10599	THIO_HUMAN	Thioredoxin
Q86V81	THOC4_HUMAN	THO complex subunit 4
Q04724	TLE1_HUMAN	Transducin-like enhancer protein 1
Q9UPN9	TRI33_HUMAN	E3 ubiquitin-protein ligase TRIM33
Q9BRA2	TXD17_HUMAN	Thioredoxin domain-containing protein 17
Q93009	UBP7_HUMAN	Ubiquitin carboxyl-terminal hydrolase 7
P55060	XPO2_HUMAN	Exportin-2 (Exp2)
Q96MM3	ZFP42_HUMAN	Zinc finger protein 42 homolog
O75362	ZN217_HUMAN	Zinc finger protein 217
