# Supplementary material for: ELF5 modulates the estrogen receptor cistrome in breast cancer
Source: PLoS Genet. 2020 Jan 2;16(1):e1008531. doi: 10.1371/journal.pgen.1008531 (PMC6959601; doi:10.1371/journal.pgen.1008531)
Supplement: S3 Table — Comparison of proteins found to interact with ELF5 by RIME in breast cancer cells compared to those identified as interacting with ELF5 [49] in mouse trophoblast stem cells by mass spectrometry (MS). (PDF) [file pgen.1008531.s012.pdf]

**Supplementary Table 3: Common proteins identified in ELF5-V5 RIME (MCF7 cells) and Elf5 MS (mouse trophoblastic stem cells)**

| **Accession** | **Uniprot ID** | **Protein Name** |
|---|---|---|
| Q12830 | BPTF_HUMAN | Nucleosome-remodeling factor subunit BPTF |
| Q9UKW6 | ELF5_HUMAN | ETS-related transcription factor Elf-5 |
| Q6ISB3 | GRHL2_HUMAN | Grainyhead-like protein 2 homolog |
| P16403 | H12_HUMAN | Histone H1.2 |
| P51610 | HCFC1_HUMAN | Host cell factor 1 |
| P20042 | IF2B_HUMAN | Eukaryotic translation initiation factor 2 subunit 2 |
| P52292 | IMA1_HUMAN | Importin subunit alpha-1 |
| O60341 | KDM1A_HUMAN | Lysine-specific histone demethylase 1A |
| Q9UQ80 | PA2G4_HUMAN | Proliferation-associated protein 2G4 |
| O75400 | PR40A_HUMAN | Pre-mRNA-processing factor 40 homolog A |
| Q02543 | RL18A_HUMAN | 60S ribosomal protein L18a |
| P62888 | RL30_HUMAN | 60S ribosomal protein L30 |
| P46777 | RL5_HUMAN | 60S ribosomal protein L5 |
| P62841 | RS15_HUMAN | 40S ribosomal protein S15 |
| P50990 | TCPQ_HUMAN | T-complex protein 1 subunit theta |
| Q02880 | TOP2B_HUMAN | DNA topoisomerase 2-beta |
