# Supplementary material for: ELF5 modulates the estrogen receptor cistrome in breast cancer
Source: PLoS Genet. 2020 Jan 2;16(1):e1008531. doi: 10.1371/journal.pgen.1008531 (PMC6959601; doi:10.1371/journal.pgen.1008531)
Supplement: S1 Document — Full description of methods used in PDF format. (DOCX) [file pgen.1008531.s013.docx]

## Full Materials and Methods

**Stable cell lines and culture**

ELF5 Isoform 2 cell lines were created and maintained as per [1]. Puromycin (Sigma-Aldrich, St Louis, Missouri, USA) was added at a concentration of 1ug/mL to maintain selection pressure. Doxycycline (Dox, Sigma-Aldrich) was added at a concentration of 0.1ug/mL daily to induce ELF5 protein expression (vehicle control = water).

**ChIP-seq**

MCF7-ELF5-Isoform2-V5 cells were seeded in 15cm plates, and doxycycline (or vehicle) treatment was commenced 24 hours after plating. After 48 hours of doxycycline treatment, cells were cross-linked for 10 minutes at room temperature using 1% formaldehyde diluted in cell growth medium. After 10 minutes, formaldehyde was quenched with 0.2M glycine. Plates were then placed on ice and washed x 2 with cold PBS. Cross-linked cells were collected in 2mL PBS using a cell scraper and pellets containing approximately 20 million cells were stored at -70oC. Four independent replicates for ER, FOXA1, ELF5-V5 and H3K4me3 ChIP-seqs and 6 input replicates (one per lane) were performed according to the protocols described in [2] and [1], with or without Dox treatment. ELF5-V5 samples were treated with Dox only.

**ChIP-seq peak calling**

Peaks were called using MACS 2.0.10 with parameters --keepdup all, -B, --q 0.01 and option “--broad” for H3K4me3 [4]. In order to define consensus peaks among the replicates, genomic regions of peak overlaps were first calculated with R (v.3.4.2.) using function *reduce* from package GenomicRanges [5], and those that were present in three out of four replicates (or two out of two for H3K4me3) were used in further analysis. Statistical significance of differential binding to peak regions was defined with R package DiffBind with default parameters [6]. QC metrics such as read depth, quality scores and GC content and enrichment for genomic regions (promoters, UTRs etc) were retrieved with R packages ChIPQC [7] and ChIPpeakAnno [8].

**Functional annotation of peak regions**

Functional analyses of ChIP-seq data were performed using Genomic Regions Enrichment of Annotations Tool (GREAT) [9]. Gene regulatory domains, as defined by 5.0kb upstream and 1.0kb downstream of TSS, were extended in both directions to the nearest gene but no more than the maximum extension (1000kb) in one direction. Statistical significance of enriched terms was assessed using the binomial test over genomic regions. Additional functional analyses of the ELF5 ChIP-seq data were performed using Enrichr [10, 11] with top 2000 peaks, sorted by MACS score. Enriched ChIP sets for top 100 differentially expressed genes were defined by ChEA tool over with the FDR of 0.05 [11]. Enrichment of transcription factor DNA binding motifs under peaks was performed with MEME-ChIP, using default parameters [12]. Significance of overlaps between ChIP-seq peaks was assessed with function  *peakPermTest*  from R package ChIPpeakAnno, with 100000 permutation [8].

**Analysis of repeat regions**

Statistical significance of overlap between peaks and repeat regions was assessed with log odds ratios. Log odds ratios are calculated from contingency tables of Fisher’s exact test for overlap between a region of interest and annotation whose enrichment is being tested. More specifically, if we are looking at a region P (a set of ChIP peaks) and overlap with annotation A (a set of repetitive elements of the same type), then *a* is the number of nucleotides of overlap between P and A, b is the number of nucleotides in annotation A without *a, c* is the number of nucleotides in P that do not contain A, and *d* is the total number of genomic nucleotides without P and A. The odds ratio is then given as (*a/b*)*/*(*c/d*). Confidence intervals for log odds ratio are then calculated as 1.96 times standard error, which is given as a square root of 1/*a*+1/*b*+1/*c*+1/*d*.

**Annotation databases**

GREAT functional enrichments were performed on MSigDB v6.0 GO Biological Process and Hallmark Collection gene sets [13]. Additional functional annotation of DE genes and RIME candidate proteins was performed with the Database for Annotation, Visualization and Integrated Discovery (DAVID) v6.8 [14, 15]. MCF-7 enhancer and superenhancer regions were defined as reported in [16]. Genomic regions with repetitive elements were adopted from Repbase [17].

**ChIP-Seq data**

The ChIP-seq data was assembled from these publications [18-33] for the comparison with ELF5 in Figure 1. Supplementary Document2, MCF7_ChIP_data.csv contains the links to the data sets.

**Data analysis and visualization**

ChIP-seq data was visualised using Integrative Genomics Viewer [34, 35]. Tracks of ChromHMM chromatin states [36] for MCF7 cells was adopted from [37]. The transcription factor binding intensity heatmaps were generated using R package ChIPseeker [38] and deepTools [39]. Correlation analyses were performed in GraphPad (Prism) using Spearman rank-order or Pearson correlation as indicated. Venn diagrams were created using online software (http://bioinformatics.psb.ugent.be/webtools/Venn/) or BioVenn [40]. Word clouds for enriched pathways were generated online (http://www.wordclouds.com), with word size proportional to the number of occurrences (minimum 2).

**RNA-sequencing (MCF7-ELF5-V5 cells)**

MCF7-ELF5-Isoform2-V5 cells were seeded in 15cm plates, and doxycycline (or vehicle) treatment was commenced 24 hours after plating. Cell pellets were collected after 48 hours of doxycycline treatment and stored at -70oC. RNA was extracted from thawed pellets with phenol/chloroform using the miRNeasy Mini Kit (Qiagen) with on-column DNase treatment. RNA quality was assessed on the 2100 Bioanalyser using RNA Nano chips (Agilent), with all samples having an RNA Integrity Number (RIN) of 9.8-9.9 (out of 10). RNA samples were submitted to The Ramaciotti Centre for Gene Function Analysis (UNSW, Sydney) for sequencing. Samples were prepared with the TruSeq Stranded Total RNA Sample prep kit (RS-122-2201 Illumina, San Diego, California, USA) according to the manufacturer’s instructions. One ug of total RNA was used as input to the ribosomal ribozero RNA depletion, followed by 13 cycles of PCR to amplify the adapter-ligated cDNA. All 6 samples (three -Dox and three +Dox experimental replicates) were pooled in one lane.  Sequencing was performed on the Illumina HiSeq2000 using v3 SBS reagents and 100bp paired-end reads. Demultiplexing of the samples was done with Casava 1.8.2 (Illumina).

**MCF7-ELF5-V5 RNA-sequencing analysis**

Sequences were trimmed for adapters and quality using Fastq-Mcf. Alignment was done with STAR (v 2.4.0d) [41] against the human genome (hg38) with gencode v20 annotations. Transcript counts were summarised and transcripts per million (TPM) calculated using RSEM (v 1.2.18) [42]. Counts were normalised using TMM [43] and transformed using voom [44]. Differential expression analysis was carried out using limma [45]. Alignment and differential expression analysis were performed by Dr Daniel Roden (Garvan Institute of Medical Research).

Gene Set Enrichment Analysis (GSEA) [46] was run in GenePattern [47] in pre-ranked mode using a ranked list of the LIMMA moderated t-statistics. One thousand gene-set permutations were performed using minimum and maximum gene-set sizes of 15 and 500, respectively. Gene-sets used in GSEA were extracted from version 6.0 of the Broad Institute’s Molecular Signatures Database (MSigDB) [13] and extended with additional curated gene-sets from literature, previously used for ELF5 microarray analysis [1]. Network-based visualization and analysis of the GSEA results was carried out using the Cytoscape [48] Enrichment Map [49] plug-in, with thresholds of: FDR (Q-value) = 0.05 (or 0.10 where indicated); p-value = 0.005; and overlap coefficient cutoff = 0.5. Gene set clusters were manually annotated with functional themes.

**Rapid Immunoprecipitation of Endogenous Protein (RIME)**

MCF7-ELF5-Isoform2-V5 cells were grown in multiple 15cm plates (Corning), treated with doxycycline for 72 hours, then cross-linked using 1% methanol-free formaldehyde (Thermo Fisher) diluted in serum-free RPMI medium. After 10 minutes, formaldehyde was quenched with 0.2M glycine. Plates were then placed on ice and washed x 2 with cold PBS. 1mL of PBS containing calcium and magnesium salts (Thermo Fisher) and Complete EDTA-free Protease Inhibitor Cocktail (Roche, Basel, Switzerland) was added to the cells, which were then collected using a cell scraper. Cells were pooled and pellets containing approximately 50 million or 100 million cells were stored at -70^o^C.

RIME was performed using cross-linked cell pellets according to the previously published protocol with some modifications [29]. For RIME experiment 1, cross-linked cell pellets were shipped to the laboratory of Dr Jason Carroll (Cancer Research UK Cambridge Institute), and ELF5-V5 RIME was performed as per the published protocol using a 1:1 mix of anti-ELF5 and anti-V5 antibodies (see below). RIME experiments 2-5 were performed in collaboration with Drs Mark Molloy and Christoph Krisp at the Australian Proteome Analysis Facility (APAF, Macquarie University, Sydney). Cell pellets x 2 (100 million for experiments 2-4 and 50 million for experiment 5) were thawed on ice and resuspended in 10mL of LB1 buffer (50mM HEPES-KOH pH 7.5, 140mM NaCl, 1mM EDTA, 10% glycerol, 0.5% Igepal CA-630, and 0.25% Triton X-100) with protease inhibitors (Roche Complete Protease Inhibitor cocktail, with added Mg132, sodium vanadate and dithiothreitol). The cells were rotated at 4^o^C for 30-60 minutes, pelleted, and the supernatant (cytoplasmic fraction) removed. Cells were resuspended in 10mL of LB2 buffer (10mM Tris-HCl pH 8.0, 200mM NaCl, 1mM EDTA, 0.5mM EGTA, protease inhibitors), rotated at 4^o^C for 30 minutes, and pelleted. Finally, cells were resuspended in 6mL of LB3 buffer (10mM Tris-HCl pH 8.0, 100mM NaCl, 1mM EDTA, 0.5mM EGTA, 0.1% sodium deoxycholate, 0.5% N-lauroylsarcosine, protease inhibitors) and the two samples pooled.

The pooled sample was then aliquoted into microcentrifuge tubes (300uL per tube) and sonicated using a probe sonicator (4 x 10 second cycles on ice). Triton X-100 (10%) diluted in LB3 was added to each sample tube (30uL), and the lysates were centrifuged for at 20,000 rcf for 10 minutes at 4^o^C to purify debris. The supernatants from each tube were then re-pooled. A small volume (~50uL) was put aside for subsequent western blot analysis of input protein, as well as reverse cross-linking, DNA purification, and agarose gel analysis of fragment sizes. The sample was then divided into 2 x 15mL tubes and incubated on a rotator overnight at 4^o^C with Pierce protein A/G magnetic beads (Thermo Fisher). The beads (110uL per immunoprecipitation) were pre-bound with a 1:1 combination of either 10ug anti-V5 (R-960-25, Thermo Fisher) and 10ug anti-ELF5 N-20 (sc-9645, SCBT) primary antibodies, or equivalent amounts of mouse IgG2a (Dako X0943, Agilent) and goat IgG (sc-2028, SCBT) isotype control antibodies. Following the overnight incubation, the beads were washed 5 times in 1mL of RIPA buffer, with approximately 10% of the sample reserved for western blot analysis after the final wash. The remaining beads were washed twice in 100mM ammonium hydrogen carbonate (AMBIC) solution, with the sample transferred to a new microcentrifuge tube after the first AMBIC wash. The beads were then resuspended in 50uL AMBIC solution and delivered to APAF, where the remainder of the protocol was performed by Christoph Krisp. Beads were diluted with 100 µL of 100mM triethylammonium bicarbonate and 1% sodium deoxycholate and boiled at 95^o^C for 5 minutes. After disulfate bond reduction with 10 mM dithiotreitol (30 minutes at 60^o^C) and cysteine alkylation with 20 mM Iodoacetamide (30 minutes at 37^o^C in the dark), each sample was digested with trypsin overnight at 37^o^C (about 20:1 protein to enzyme). Supernatant was removed from the beads and transferred to a new tube. Samples were acidified with formic acid (1% final concentration) to quench digestion and precipitate sodium deoxycholate. Samples were then spun at 14,000 rcf for 5 min and the supernatant transferred to a new tube. Samples were dried in a vacuum concentrator and resuspended in 20 µL 2% acetonitrile (ACN) and 0.1% formic acid.

Next, 10uL of each sample was injected onto a C18 reversed phase (RP) peptide trap chip (0.5 mm, 200 µm, 300Å ChromXP C18 RP) for purification. Peptides were eluted from the trap and separated on a 15 cm chip column (200 µm, 300Å ChromXP C18 RP) using a linear solvent gradient from 5% ACN 0.1% formic acid to 40% ACN, 0.1% formic acid at 600nL/min over a 60 min period. The LC eluents were subject to positive ion nanoflow electrospray MS analysis in an information dependant acquisition mode (IDA) on a 5600 TripleToF mass spectrometer with Eksigent NanoLC Ultra with cHiPLC system (SCIEX, Framingham, Massachusetts, USA).

In information dependent acquistion (IDA) mode, a TOF-MS survey scan was acquired (m/z 350-1500, 250 ms accumulation time), then the 20 most intense multiply charged ions (2+ - 4+; counts >150cps) in the survey scan were sequentially subjected to MS/MS analysis. MS/MS spectra were accumulated for 100 ms (m/z 100-1500) with rolling collision energy. IDA data were searched against the Human SwissProt data base release April 2014 with ProteinPilot software version 4.2 (SCIEX) using the mascot algorithm. Decoy database search was enabled to allow false discovery rate (FDR) calculation. Proteins accepted to be present in a sample had a FDR < 1%.

**Co-immunoprecipitations for western blot**

ELF5/V5 co-immunoprecipitations (shown in Figure 5.5) were prepared according to the protocol described above. After the final RIPA buffer wash, approximately 10% of the IP beads were pelleted and stored at -20^o^C. For western blot analysis, the beads were resuspended in 20uL of NuPAGE Sample Buffer containing 2x Reducing Agent (Thermo Fisher) and incubated at 95^o^C for 5 minutes. The supernatant was then transferred to a fresh tube and run on a pre-cast 4-12% Bis-Tris polyacrylamide gel (Thermo Fisher).

**Western blots**

Western blots were performed according to the following general protocol; more specific information is provided in Tables 2.2 (experimental details) and 2.3 (antibody details). Whole cell lysates were prepared from adherent cells or cell pellets using Normal Lysis Buffer (1.2% HEPES, 1% Triton X-100, 10% glycerol, 0.8% NaCl, 0.03% MgCl2, 0.04% EGTA, 1.0% disodium pyrophosphate, 0.4% NaF), except where otherwise indicated. Roche Complete EDTA-free Protease Inhibitor Cocktail and PhosSTOP phosphatase inhibitors (for phospho-blot samples) were added to the lysis buffer. Cells were incubated on ice, vortex, and centrifuged at 10,000rpm for 10 minutes at 4^o^C; supernatant was collected and stored at -70^o^C. Protein concentration was measured using the Bio-Rad Protein Assay (Bio-Rad Laboratories, Hercules, California, USA). Cell lysate samples were prepared using NuPAGE Sample Buffer and Reducing Agent (Thermo Fisher), heated at 70^o^C for 10 minutes, and run on pre-cast NuPAGE gels (Thermo Fisher) in MOPS buffer or MES buffer (for selected ELF5 blots). Tris-acetate gels (phospho-DNA-PKcs antibodies, Figure 5.21B) were run in Tris-Acetate buffer (50mM tricine, 50mM tris base, 0.1% SDS). Proteins were transferred to polyvinylidene difluoride (PVDF) membrane at 100V for 1 hour (increased to 2 hours for selected blots examining DNA-PKcs due to its large size). Membranes were cut at specific molecular weights, guided by Precision Plus Protein Dual-Colour Standards (Bio-Rad Laboratories), to facilitate incubations with multiple blocking solutions and primary antibodies. Membranes were blocked for 1-2 hours at room temperature in TBS-tween (10mM Tris base, 150mM NaCl, 0.1% Tween) with either 5% skim milk, 5% bovine serum albumin (BSA, Sigma-Aldrich, phospho-ER and phospho-DNA-PKcs membranes), or 5% donkey serum (Jackson ImmunoResearch, ELF5 N-20 membranes). Primary antibody incubation was performed overnight at 4^o^C with gentle shaking. Membranes were then incubated with secondary HRP-conjugated antibody diluted 1:2,000-1:5,000 in blocking solution for 1 hour at room temperature with gentle shaking. TBS-tween washes were performed after each antibody incubation. Proteins were detected using enhanced chemiluminescence solution (Western Lightning Plus, Perkin Elmer, Waltham, Massachusetts, USA) and x-ray film (Fujifilm, Tokyo, Japan).

**Proximity ligation assays (PLAs)**

Cells were seeded on glass coverslips in 12-well plates (Corning) and treated with doxycycline or vehicle for 48 hours. Three biological replicates were performed. Coverslips were washed x 2 in room temperature Dulbecco’s phosphate-buffered saline (PBS, Thermo Fisher), fixed for 10 minutes with 4% PFA diluted in PHEM buffer (see above), permeabilised for 10 minutes with 0.5% Triton X-100, and blocked with 10% donkey serum for 2 hours at 37^o^C (Jackson ImmunoResearch). Primary antibody incubation was conducted overnight at 4^o^C using the following antibodies (or combinations of antibodies) diluted in 10% donkey serum/PHEM solution: anti-V5 1:1000 (#13202, CST), anti-DNA-PKcs 1:50 (#12311, CST), anti-DNA-PKcs 1:1000 (MS-423-P1, Thermo Fisher). Isotype control antibodies were diluted to ensure equivalent amounts of antibody to matched primary: Mouse IgG1 (Dako X0931, Agilent, Santa Clara, California, USA), Mouse IgG2a (Dako X0943, Agilent), Rabbit IgG (NB810-56910, Novus Biologicals, Littleton, Colorado, USA). On day 2, the standard Duolink protocol was followed (Sigma-Aldrich, protocol summarised in Figure 5.6). Briefly, coverslips were incubated for 1 hour at 37^o^C with Duolink green minus and plus probes (rabbit and mouse), according to the species of primary antibody/antibodies used. This was followed by incubation with ligation solution (30 mins at 37^o^C) and amplification solution (100 mins at 37^o^C). Total reaction volumes were 40uL per coverslip. 4 x washes in Duolink wash buffer A (0.01M Tris, 0.15M NaCl, 0.05% Tween-20) were carried out between each step and the final 3 x washes in Duolink wash buffer B (0.2M Tris and 0.1M NaCl, undiluted or 1:100 as per protocol). Coverslips were allowed to dry in the dark and then mounted on glass slides with Duolink In Situ Mounting Medium with DAPI. Slides were stored at 4^o^C for a period of up to 3 weeks until imaging.

**PLA image acquisition**

PLA coverslips were imaged 8-18 days after completion of the Duolink staining protocol on a Leica DM5500 microscope (Leica Microsystems). To ensure unbiased image acquisition, groups of cells to be imaged were identified by horizontal movement across the coverslip for a total of 4 rows (separated by 2mm) using the DAPI (nuclear) A4 filter cube. The PLA signals were briefly viewed (using the L5 green Leica filter cube) to ensure they were in focus and the image was acquired using pre-defined exposure times. In this way, the cells for PLA quantification were selected without knowledge of the PLA signal level. Approximately 15-120 images were taken per coverslip; the total number of cells (nuclei) for the combined replicates are shown in Figure 5.10. Due to technical issues, only two (of three) experimental replicates were imaged for the V5 and DNA-PKcs CST antibody combination and controls.

**PLA image analysis**

PLA images were analysed using a FIJI [50] macro created by Andrew Law . Nuclear images were modified with ‘Enhance Contrast’ at a saturation value of 0.4, ‘Subtract Background’ with a rolling ball radius of 100, and then converted to an 8-bit greyscale image, and thresholded. They were then processed with ‘Watershed’ and ‘Analyse Particles’ to select for and create a mask image of the nuclei. Foci (PLA signals) were then selected using the ‘Find Maxima’ function and a single point mask was created from the foci selection. The mask images of the foci and nuclei were added together with the ‘Image Calculator’ function, and non-nuclear signals defined. Nuclear signals were then calculated by subtracting non-nuclear signals from the total signal number. Chi-square analysis of the signal distribution was performed using GraphPad Prism.

**siRNA transfection**

ON-TARGETplus human *PRKDC* SMART pool siRNA (Dharmacon, Lafayette, Colorado, USA) was resuspended in nuclease-free sterile water at 100uM, and stored as single-use 5uL aliquots at -70^o^C. ON-TARGETplus non-targeting siRNA #1 (Dharmacon) was used as a control. All transfections were performed using Lipofectamine RNAiMAX transfection reagent (Thermo Fisher), at 5nM siRNA (0.12uL) and 2.5uL Lipofectamine per well (6-well plate) in a total volume of 2.4mL. The siRNA and Lipofectamine mixture was diluted in Opti-MEM (Thermo Fisher) and incubated at room temperature for approximately 20 minutes. 400uL of siRNA/Lipofectamine mixture was then added to each well (or Opti-MEM only for the untransfected control), followed by 2.0mL of cells suspended in normal medium. Cell numbers for 6-well plates (Corning Life Sciences, Tewksbury, Massachusetts, USA) were 80,000 cells/well (MCF7 lines), 150,000 cells/well (T47D lines), or 40,000 cells/well (MDA-MB-231 lines). No antibiotics, including puromycin, were added during transfection. After 24 hours, the medium was changed and puromycin was commenced to maintain doxycycline-induced ELF5 expression. Doxycycline treatment was started on day 2 and cells were collected on day 4.

**End-point PCR**

RNA was extracted using the RNeasy Mini Kit with DNase treatment (Qiagen). cDNA was made from 2ug RNA using the Applied Biosystems High Capacity cDNA Reverse Transcription Kit (Thermo Fisher) with RNasin Ribonuclease Inhibitor (Promega, Madison, Wisconsin, USA). PCR reactions were run for 25 cycles using the PCR Reagent System (Thermo Fisher) with optimised annealing temperatures and magnesium concentrations. *ELF5* Isoform 2/3 primers designed using NCBI Primer-BLAST (5’ to 3’): AGCGCCTGCCTTCTCTTGCC (forward) and CCCCACATCTTTGCCAGGGCTT (reverse). Amplicons were visualised on a 1% agarose/ethidium bromide gel (Figure 3.12B).

**Quantitative PCR**

RNA was extracted using the RNeasy Mini Kit with DNase treatment (Qiagen) and quantified using the Nanodrop spectrophotometer (Thermo Fisher). cDNA was made using the Applied Biosystems High Capacity cDNA Reverse Transcription Kit Thermo Fisher) with RNasin Ribonuclease Inhibitor (Promega). All qPCR reactions were run on the Applied Biosystems ABI7900 qPCR machine (Thermo Fisher). Two to three technical replicates were run for each sample, as well as negative controls (no template, no reverse transcriptase, water). Standard curves using a 1:10 dilution series were run for every assay to determine amplification efficiency and relative quantity.

Taqman assays were run using 4.5uL cDNA (diluted 1:5-1:10 in nuclease-free water) and 5.5uL assay (diluted 1:11 in Taqman Gene Expression Mastermix, Thermo Fisher) using standard Taqman cycling conditions. Roche Universal Probe Library (UPL) assays were designed using the online Roche ProbeFinder software. All Roche assays were tested prior to use with a 6-point 1:10 dilution series and assays with poor amplification were not used. Each 10uL Roche qPCR reaction included 0.4uL forward primer (10uM), 0.4uL reverse primer (10uM), 0.1uL UPL probe, 5uL LightCycler 480 Probes Master reaction mix (Roche) and 4.1uL of diluted cDNA. Reactions were run in 384-well plates on the ABI7900 qPCR machine (Life Technologies) using the Roche UPL protocol (denature 94^o^C for 10 mins, cycle 94^o^C for 15 sec/60^o^C for 30 sec/72^o^C for 15sec (x45), cooling 40^o^C for 2 mins).

Results were analysed using SDS 2.4 (Thermo Fisher) and qbase+ software (Biogazelle, Gent, Belgium) [51]. For the Chapter 3 qPCR panel (Figure 3.17G), paired *t* tests were used to calculate *p-*values, comparing -dox and +dox samples (3-4 pairs per cell line group). Correction for multiple comparisons was performed using the Benjamini-Hochberg method [52]. For the Chapter 5 qPCR panel (Figure 5.18), statistical analysis was performed using qbase+ one-way ANOVA with correction for multiple hypotheses.

**Bioinformatic techniques**

For the ELF5 ChIP-seq, a probable list of direct target genes was generated by: (i) Assigning each ChIP-seq peak to the closest gene; (ii) Filtering to include only peaks where the closest gene is within 10kb of the transcription start; (iii) Overlap of this gene list with differentially expressed genes identified in the ELF5 RNA-seq, defined by FDR <0.05 and absolute fold-change >1.5. For the FOXA1 peaks gained or lost on ELF5 over-expression, lists of potential target genes were generated using GREAT (described above).

Differentially expressed gene lists were analysed for functional enrichments using MSigDB v6.0 GO Biological Process and Hallmark Collection gene sets [13]. RIME candidate proteins were analysed using the Database for Annotation, Visualization and Integrated Discovery (DAVID) v6.8 [14, 15].

Gene identifiers (including Affy probe IDs, Ensembl gene IDs, and HGNC symbols) were converted using Ensembl Biomart [53] and the DAVID gene ID conversion tool.

Correlation analyses were performed in GraphPad (Prism) using Spearman rank-order or Pearson correlation as indicated.

Venn diagrams were created using online software (http://bioinformatics.psb.ugent.be/webtools/Venn/) or BioVenn [40]. Wordclouds for enriched pathways were generated online (http://www.wordclouds.com), with word size proportional to the number of occurrences (minimum 2).

**References**

1. Kalyuga M, Gallego-Ortega D, Lee HJ, Roden DL, Cowley MJ, Caldon CE, Stone A, Allerdice SL, Valdes-Mora F, Launchbury R, et al: **ELF5 Suppresses Estrogen Sensitivity and Underpins the Acquisition of Antiestrogen Resistance in Luminal Breast Cancer.** *PLoS Biol* 2012, **10:**e1001461.

2. Hurtado A, Holmes KA, Ross-Innes CS, Schmidt D, Carroll JS: **FOXA1 is a key determinant of estrogen receptor function and endocrine response.** *Nat Genet* 2011, **43:**27-33.

3. Li H, Durbin R: **Fast and accurate long-read alignment with Burrows-Wheeler transform.** *Bioinformatics* 2010, **26:**589-595.

4. Zhang Y, Liu T, Meyer CA, Eeckhoute J, Johnson DS, Bernstein BE, Nusbaum C, Myers RM, Brown M, Li W, Liu XS: **Model-based analysis of ChIP-Seq (MACS).** *Genome Biol* 2008, **9:**R137.

5. Lawrence M, Huber W, Pages H, Aboyoun P, Carlson M, Gentleman R, Morgan MT, Carey VJ: **Software for computing and annotating genomic ranges.** *PLoS Comput Biol* 2013, **9:**e1003118.

6. Ross-Innes CS, Stark R, Teschendorff AE, Holmes KA, Ali HR, Dunning MJ, Brown GD, Gojis O, Ellis IO, Green AR, et al: **Differential oestrogen receptor binding is associated with clinical outcome in breast cancer.** *Nature* 2012, **481:**389-393.

7. Carroll TS, Liang Z, Salama R, Stark R, de Santiago I: **Impact of artifact removal on ChIP quality metrics in ChIP-seq and ChIP-exo data.** *Front Genet* 2014, **5:**75.

8. Zhu LJ, Gazin C, Lawson ND, Pages H, Lin SM, Lapointe DS, Green MR: **ChIPpeakAnno: a Bioconductor package to annotate ChIP-seq and ChIP-chip data.** *BMC Bioinformatics* 2010, **11:**237.

9. McLean CY, Bristor D, Hiller M, Clarke SL, Schaar BT, Lowe CB, Wenger AM, Bejerano G: **GREAT improves functional interpretation of cis-regulatory regions.** *Nat Biotechnol* 2010, **28:**495-501.

10. Chen EY, Tan CM, Kou Y, Duan Q, Wang Z, Meirelles GV, Clark NR, Ma'ayan A: **Enrichr: interactive and collaborative HTML5 gene list enrichment analysis tool.** *BMC Bioinformatics* 2013, **14:**128.

11. Kuleshov MV, Jones MR, Rouillard AD, Fernandez NF, Duan Q, Wang Z, Koplev S, Jenkins SL, Jagodnik KM, Lachmann A, et al: **Enrichr: a comprehensive gene set enrichment analysis web server 2016 update.** *Nucleic Acids Res* 2016, **44:**W90-97.

12. Machanick P, Bailey TL: **MEME-ChIP: motif analysis of large DNA datasets.** *Bioinformatics* 2011, **27:**1696-1697.

13. Liberzon A, Birger C, Thorvaldsdóttir H, Ghandi M, Mesirov Jill P, Tamayo P: **The Molecular Signatures Database Hallmark Gene Set Collection.** *Cell Systems* 2015, **1:**417-425.

14. Huang da W, Sherman BT, Lempicki RA: **Systematic and integrative analysis of large gene lists using DAVID bioinformatics resources.** *Nat Protoc* 2009, **4:**44-57.

15. Huang da W, Sherman BT, Lempicki RA: **Bioinformatics enrichment tools: paths toward the comprehensive functional analysis of large gene lists.** *Nucleic Acids Res* 2009, **37:**1-13.

16. Cao F, Fang Y, Tan HK, Goh Y, Choy JYH, Koh BTH, Hao Tan J, Bertin N, Ramadass A, Hunter E, et al: **Super-Enhancers and Broad H3K4me3 Domains Form Complex Gene Regulatory Circuits Involving Chromatin Interactions.** *Sci Rep* 2017, **7:**2186.

17. Bao W, Kojima KK, Kohany O: **Repbase Update, a database of repetitive elements in eukaryotic genomes.** *Mob DNA* 2015, **6:**11.

18. Barutcu AR, Hong D, Lajoie BR, McCord RP, van Wijnen AJ, Lian JB, Stein JL, Dekker J, Imbalzano AN, Stein GS: **RUNX1 contributes to higher-order chromatin organization and gene regulation in breast cancer cells.** *Biochim Biophys Acta* 2016, **1859:**1389-1397.

19. Cheng D, Vemulapalli V, Lu Y, Shen J, Aoyagi S, Fry CJ, Yang Y, Foulds CE, Stossi F, Trevino LS, et al: **CARM1 methylates MED12 to regulate its RNA-binding ability.** *Life Sci Alliance* 2018, **1:**e201800117.

20. Consortium EP: **An integrated encyclopedia of DNA elements in the human genome.** *Nature* 2012, **489:**57-74.

21. Elster D, Tollot M, Schlegelmilch K, Ori A, Rosenwald A, Sahai E, von Eyss B: **TRPS1 shapes YAP/TEAD-dependent transcription in breast cancer cells.** *Nat Commun* 2018, **9:**3115.

22. Fournier M, Bourriquen G, Lamaze FC, Cote MC, Fournier E, Joly-Beauparlant C, Caron V, Gobeil S, Droit A, Bilodeau S: **FOXA and master transcription factors recruit Mediator and Cohesin to the core transcriptional regulatory circuitry of cancer cells.** *Sci Rep* 2016, **6:**34962.

23. Jozwik KM, Chernukhin I, Serandour AA, Nagarajan S, Carroll JS: **FOXA1 Directs H3K4 Monomethylation at Enhancers via Recruitment of the Methyltransferase MLL3.** *Cell Rep* 2016, **17:**2715-2723.

24. Kittler R, Zhou J, Hua S, Ma L, Liu Y, Pendleton E, Cheng C, Gerstein M, White KP: **A comprehensive nuclear receptor network for breast cancer cells.** *Cell Rep* 2013, **3:**538-551.

25. Liu Z, Merkurjev D, Yang F, Li W, Oh S, Friedman MJ, Song X, Zhang F, Ma Q, Ohgi KA, et al: **Enhancer activation requires trans-recruitment of a mega transcription factor complex.** *Cell* 2014, **159:**358-373.

26. Lo R, Matthews J: **High-resolution genome-wide mapping of AHR and ARNT binding sites by ChIP-Seq.** *Toxicol Sci* 2012, **130:**349-361.

27. Magnani L, Ballantyne EB, Zhang X, Lupien M: **PBX1 genomic pioneer function drives ERalpha signaling underlying progression in breast cancer.** *PLoS Genet* 2011, **7:**e1002368.

28. Magnani L, Stoeck A, Zhang X, Lanczky A, Mirabella AC, Wang TL, Gyorffy B, Lupien M: **Genome-wide reprogramming of the chromatin landscape underlies endocrine therapy resistance in breast cancer.** *Proc Natl Acad Sci U S A* 2013, **110:**E1490-1499.

29. Mohammed H, D'Santos C, Serandour AA, Ali HR, Brown GD, Atkins A, Rueda OM, Holmes KA, Theodorou V, Robinson JL, et al: **Endogenous purification reveals GREB1 as a key estrogen receptor regulatory factor.** *Cell Rep* 2013, **3:**342-349.

30. Pugacheva EM, Rivero-Hinojosa S, Espinoza CA, Mendez-Catala CF, Kang S, Suzuki T, Kosaka-Suzuki N, Robinson S, Nagarajan V, Ye Z, et al: **Comparative analyses of CTCF and BORIS occupancies uncover two distinct classes of CTCF binding genomic regions.** *Genome Biol* 2015, **16:**161.

31. Venkataraman A, Yang K, Irizarry J, Mackiewicz M, Mita P, Kuang Z, Xue L, Ghosh D, Liu S, Ramos P, et al: **A toolbox of immunoprecipitation-grade monoclonal antibodies to human transcription factors.** *Nat Methods* 2018, **15:**330-338.

32. Yang Y, Lu Y, Espejo A, Wu J, Xu W, Liang S, Bedford MT: **TDRD3 is an effector molecule for arginine-methylated histone marks.** *Mol Cell* 2010, **40:**1016-1023.

33. Zwart W, Theodorou V, Kok M, Canisius S, Linn S, Carroll JS: **Oestrogen receptor-co-factor-chromatin specificity in the transcriptional regulation of breast cancer.** *EMBO J* 2011, **30:**4764-4776.

34. Robinson JT, Thorvaldsdottir H, Winckler W, Guttman M, Lander ES, Getz G, Mesirov JP: **Integrative genomics viewer.** *Nat Biotech* 2011, **29:**24-26.

35. Thorvaldsdóttir H, Robinson JT, Mesirov JP: **Integrative Genomics Viewer (IGV): high-performance genomics data visualization and exploration.** *Briefings in Bioinformatics* 2013, **14:**178-192.

36. Ernst J, Kellis M: **ChromHMM: automating chromatin-state discovery and characterization.** *Nat Meth* 2012, **9:**215-216.

37. Taberlay PC, Statham AL, Kelly TK, Clark SJ, Jones PA: **Reconfiguration of nucleosome-depleted regions at distal regulatory elements accompanies DNA methylation of enhancers and insulators in cancer.** *Genome Res* 2014, **24:**1421-1432.

38. Holmes KA, Hurtado A, Brown GD, Launchbury R, Ross-Innes CS, Hadfield J, Odom DT, Carroll JS: **Transducin-like enhancer protein 1 mediates estrogen receptor binding and transcriptional activity in breast cancer cells.** *Proc Natl Acad Sci U S A* 2012, **109:**2748-2753.

39. Ramirez F, Dundar F, Diehl S, Gruning BA, Manke T: **deepTools: a flexible platform for exploring deep-sequencing data.** *Nucleic Acids Res* 2014, **42:**W187-191.

40. Hulsen T, de Vlieg J, Alkema W: **BioVenn – a web application for the comparison and visualization of biological lists using area-proportional Venn diagrams.** *BMC Genomics* 2008, **9:**488.

41. Dobin A, Davis CA, Schlesinger F, Drenkow J, Zaleski C, Jha S, Batut P, Chaisson M, Gingeras TR: **STAR: ultrafast universal RNA-seq aligner.** *Bioinformatics* 2013, **29:**15-21.

42. Li B, Dewey CN: **RSEM: accurate transcript quantification from RNA-Seq data with or without a reference genome.** *BMC Bioinformatics* 2011, **12:**323.

43. Robinson MD, Oshlack A: **A scaling normalization method for differential expression analysis of RNA-seq data.** *Genome Biol* 2010, **11:**R25.

44. Law CW, Chen Y, Shi W, Smyth GK: **voom: Precision weights unlock linear model analysis tools for RNA-seq read counts.** *Genome Biol* 2014, **15:**R29.

45. Smyth GK: **Linear models and empirical bayes methods for assessing differential expression in microarray experiments.** *Stat Appl Genet Mol Biol* 2004, **3:**Article3.

46. Subramanian A, Tamayo P, Mootha VK, Mukherjee S, Ebert BL, Gillette MA, Paulovich A, Pomeroy SL, Golub TR, Lander ES, Mesirov JP: **Gene set enrichment analysis: a knowledge-based approach for interpreting genome-wide expression profiles.** *Proc Natl Acad Sci U S A* 2005, **102:**15545-15550.

47. Reich M, Liefeld T, Gould J, Lerner J, Tamayo P, Mesirov JP: **GenePattern 2.0.** *Nat Genet* 2006, **38:**500-501.

48. Shannon P, Markiel A, Ozier O, Baliga NS, Wang JT, Ramage D, Amin N, Schwikowski B, Ideker T: **Cytoscape: a software environment for integrated models of biomolecular interaction networks.** *Genome Res* 2003, **13:**2498-2504.

49. Merico D, Isserlin R, Stueker O, Emili A, Bader GD: **Enrichment Map: A Network-Based Method for Gene-Set Enrichment Visualization and Interpretation.** *PLOS ONE* 2010, **5:**e13984.

50. Schindelin J, Arganda-Carreras I, Frise E, Kaynig V, Longair M, Pietzsch T, Preibisch S, Rueden C, Saalfeld S, Schmid B, et al: **Fiji: an open-source platform for biological-image analysis.** *Nat Methods* 2012, **9:**676-682.

51. Hellemans J, Mortier G, De Paepe A, Speleman F, Vandesompele J: **qBase relative quantification framework and software for management and automated analysis of real-time quantitative PCR data.** *Genome Biol* 2007, **8:**R19.

52. Benjamini Y, Hochberg Y: **Controlling the false discovery rate: a practical and powerful approach to multiple testing.** *Journal of the Royal Statistical Society Series B (Methodological)* 1995, **57:**289-300.

53. Kinsella RJ, Kahari A, Haider S, Zamora J, Proctor G, Spudich G, Almeida-King J, Staines D, Derwent P, Kerhornou A, et al: **Ensembl BioMarts: a hub for data retrieval across taxonomic space.** *Database (Oxford)* 2011, **2011:**bar030.
