# Supplementary material for: ELF5 modulates the estrogen receptor cistrome in breast cancer
Source: PLoS Genet. 2020 Jan 2;16(1):e1008531. doi: 10.1371/journal.pgen.1008531 (PMC6959601; doi:10.1371/journal.pgen.1008531)
Supplement: Attachment — Submitted filename: Response to reviewersPlOSGenetics.docx [file pgen.1008531.s015.docx]

**Response to reviewers.**

We thank the reviewers for their many comments that have produced a much improved manuscript. We have added new data to address the main concerns and we have reorganised the manuscript as suggested. This has produced a stronger and clearer case for our conclusions.

Our responses are underlined beneath the request from the reviewer.

**Reviewer #1:**

Piggin et al., present a valuable study on the role of ELF5 in mediating endocrine resistance. Several novel aspects include: 1) ELF5 ChIP-seq, 2) Insights gained from FOXA1/ER displacement and potential role in distinct gene regulation, 3) Insight into the ELF5 protein complexes. Major comments which may strengthen the findings/conclusions of this study are:

1) It’s not clear from this study whether the protein expression levels of ELF5 are consistent with expression in tumor samples upon DOX induction. My concern is whether ELF5 expression is elevated to an extent that is not observed in disease - and therefore also resulting effects on FOXA1/ER cistrome.

We have included new data (Supplementary Figure 1A) that demonstrates that the level of ELF5 induced by DOX treatment in MCF-7 cells is similar to the level of ELF5 seen during pregnancy in the mouse mammary gland. The figure also shows that in T47D cells the level of ELF5 induced by DOX treatment is below the level that can be induced by treatment of the cells with R5020. These data conclusively demonstrate that DOX does not induce ELF5 levels to super physiological levels.

2) In other disease systems, super enhancers have been used to define and predict activity of core TFs that regulate cell (disease state) identity. Are these associated with the 20+ (line 166) TFs ? or perhaps defines a distinct subset overlapping with ER-transcriptional complex members.

We are unsure about which core TFs are being referred to here. Whave added the following sentences to the manuscript. “MED1 is often used to define super enhancers and MED1 is frequently found to be a member of the 20+ set along with many members of the ER transcriptional complex such as ER, FOXA1, MLL3, GREB1, SRC2,TLE3 etc. NIPBL and Cohesin are also frequent members of this set, and are involved in enhancer-promoter communication.”

3) As FOXA1 is known to have pioneering activity, following ELF5 over-expression are de novo FOXA1 binding sites established at previously closed/open chromatin ?

We investigated this possibility and have added the sentence below. We thank the reviewer for requesting this information as it has provided a very interesting result. Using genome DNAase1 sensitivity data (Swinstead et al. new ref 38) that defined regions of open chromatin in MCF-7 cells, we found that of the 503 FOXA1 sites enriched by induction of ELF5, only 139 (28%) occurred at regions of open chromatin, indicating that most of the enriched FOXA1 sites occurred at previously closed chromatin. Thus induction of ELF5 causes FOXA1 to bind to closed chromatin, consistent with its established pioneering activity.

**Reviewer #2:**

The authors aimed to investigate the mechanism by which the alveolar lineage transcription factor ELF5 may drive endocrine therapy resistance. They use multiple ‘omics approaches to do so (with many replicates), however the data often felt like a laundry list of genes or proteins (and in some cases, only a few reproducible ones are shown) without a clear mechanism by which ELF5 acts. While the title and abstract of this paper are provoking in terms of a potential mechanism by which ELF5 can act to promote breast cancer, especially in the context of resistance to estrogen blockade, the data unfortunately fall short in actually demonstrating this…..and overall lacks a clear mechanism for which ELF5 might reorganize the epigenome, and as such, the manuscript in its current state is not sufficient for publication.

Our study set out to discover the mechanisms by which ELF5 modulates estrogen action and endocrine resistance, and we report three previously unknown mechanisms by which this occurs. They are 1, the ability of ELF5 to alter ER and FOXA1 binding to the genome in the same pattern seen when cells become resistant to antiestrogens; 2, ELF5 regulation of the expression of key ER transcription complex members; and 3, ELF5 binding directly to a number of ER transcription complex members. We believe that this is sufficiently significant to warrant publication in PLoS Genetics and reviewers 1 and 3 support this. To further define the epigenetic mechanisms of ELF5 action is a key avenue for future investigation, but this is a major undertaking and in our opinion the results would be less significant than this first report of the multitude of ways that ELF5 modulates estrogen action. To delay publishing our current findings while we spend years doing this would be detrimental to progress toward overcoming endocrine resistance.

Many of the conclusions are overstated based on what the data demonstrates, the data are largely correlative.

We have sought to clarifiy our language where the reviewer has indicated that we are overstating the conclusions, please see the specific instances below where they are mentioned by the reviewer.

1. By ChIP-seq the authors found that genomic regions of genes bound by ELF5 were also binding sites for ER and FOXA1. It is not shown anywhere what ELF5 ectopic expression looks like in either BrCa cell line… Overall, the authors need to better describe their ELF5 expression system

We have added a new supplementary figure 1A that shows the vector used to create the inducible cell lines, and a western blot is also added that shows that induction of ELF5 by DOX in MCF-7 and T47-D cells is within the level of induction produced by pregnancy in mice or treatment with R5020. These data show that DOX treatment does not produce super-physiological induction of ELF5.

Are ER and FOXA1 regulated transcriptionally by ELF5 expression? How do we know that the ER and FOXA1 new binding sites are not merely caused by transcriptional upregulation of these TFs, which in turn causes ectopic binding?

We have added a new supplementary figure (SFig. 8B and 8C), where we show that at the time points used in this study there was no change in the level of either ER or FOXA1 protein levels measured by western blot. Similarly there was no change in ER phosphorylation. Changes in ER or FOXA1 levels cannot explain their changes in binding to DNA. Ectopic binding due to increased expression of ER and FOXA1 is not possible.

, and T47D data is largely ignored throughout the manuscript.

T-47D data are used to show the effect in MCF-7 is seen in a second cell line. Since T-47D cells are order of magnitude less sensitive to estrogen than MCF-7, MCF-7 is the model of choice for the study of endocrine resistance, hence the emphasis on MCF-7.

The RNA-seq data is not shown in its own right for both cell lines. These data are critical as the authors later call ELF5 DEGs.

We now show the RNA-seq data in it own right. In the revised version we have created a new RNA-seq main figure (Fig. 2), and in a separate figure we have integrated the RNA-seq with the ChIP-seq (Fig 4).

The authors also describe overlap of ELF5 with repetitive elements, with dedicated main figures to this point, but the context of this is completely unclear as they never come back to this finding later in the paper. What is the significance of this? Does it help to understand newly acquired binding sites? (also SINE, LINE, MIRb are not clearly defined for the reader).

As the reviewer correctly points out this data is unrelated to the main aim of the paper, and so we have removed this finding from the main figures, into a new supplementary figure (SFig. 3). We state that the significance of this finding is adding ELF5 to the group of transcription factors that bind to transposable elements. This implicates ELF5 in genome evolution (V1 manuscript lines 145-148) and we have emphasised this conclusion in the revised manuscript. This is a novel and interesting discovery that we feel should be included in the manuscript.

3. They further studied ELF5 binding partners and suggested that it interacted with the ER transcription complex, such as DNA-PKcs. What does this interaction mean?

The DNA-PKcs knockdown revealed that some genes among the ELF5-regulated candidates we tested were dependent on DNA-PKcs for their expression, meaning that we have demonstrated that the physical interaction between DNA-PKcs and ELF5 also has a function. Thus ELF5 may modulate estrogen action via direct physical interaction with ER transcriptional complex members such as DNSPKcs.

It is unclear how it (DNA-PKcs) was chosen for study (besides being one of two identified through RIME)

DNA-PKcs was chosen solely as the top ranked hit from the very stringent RIME analysis. This is based on the unbiased approach offered by RIME. We deliberately did not take a candidate approach to discovery or validation.

and it was not validated by co-IP.

We add a co-IP western blots from three independent experiments (SFig. 8) that show clear co-IP of ELF5 and DNA-PKcs. We did not include these figures as we considered that the RIME and the PLA results were sufficient to demonstrate the interaction.

Also, the changes on gene expression upon its KD are not striking.

New supplementary figure B and C shows a western blot of the DNA-PKcs knockdown. These data show near total loss of DNA-PKcs. The changes seen in ELF5 regulation of VTCN1 (now also shown by western blot) and PIP as a result of the KD are very large, while others are less striking but statistically significant. Of the 8 candidates we chose based solely on ELF5 regulation, knockdown of DNA-PKcs altered the response to ELF5 in five cases. Clearly the interaction of ELF5 and DNA-PKcs is functionally important.

The authors might consider other interactors to study – e.g. splicing or replications proteins (MCMs).

We may do this in the future depending on available resources.

More generally, why did the RIME data only produce 3 proteins from 5 replicates?

The RIME experiment used 5 independent experiments carried out in 2 laboratories (Cambridge and Sydney), each with stringent cut offs and screening against libraries of known spurious interactors. Then we applied a double 5 of 5 criteria, present in all 5 test groups and absent in all 5 control groups. This is extremely stringent, resulting in 3 identified proteins. As we relaxed the stringency many more interactions were revealed (supplementary table 1). We altered the sentence describing this to emphasise how stringent our criteria are.

4. Many points are overstated in the paper based on the data presented.
-For example, the authors say at line 90 that “An ELF5 motif was present at sites of increased binding (SFig. 5C)”. Even though there is signal of ELF5 at the SFig.5C, it is not a peak or clearly biologically significant.

We have changed the sentence to more clearly represent the data, as suggested by the reviewer. The figure shows that there is an increased chance of finding an ELF5 motif beneath new FOXA1 peaks. Thus the sentence has been changed to indicate that this is the probability of an ELF5 peak. “The probability that an ELF5 motif was present at sites of new binding was increased, but not where binding was lost (SFig. 7C and D).” We do not test the biological significance of this observation, and the statement by the reviewer that it is of no biological significance cannot be justified, but the contrast to lost peaks is striking and worthy of note.

-The authors compare ELF5 expression in primary and metastatic patient samples. They state “This tumor collection contained 7 ER-positive cancers, 5 of which showed increased ELF5 expression”. This is an overstatement, since only 2 tumors showed increased in ELF5 expression. The others presented small increases at low levels that it might even be considered baseline.

We have altered the text of this section to focus on the 2 tumours that showed massively increased ELF5 expression.

Moreover, claiming that the ELF 5 signature is significant in these patients should be validated in another patient cohort in order to make such generalizations.

We are keen to do this but we cannot find another cohort of matched ER+ breast cancer primaries and metastases. We would appreciate the reviewer specifying which cohort we could use, and if no such cohort exists we ask the reviewer to please withdraw this request if it is impossible to fulfil.

-The authors also overstate the effects of ELF5 induction in some of key genes analyzed in Supp. Fig. 10, e.g. GRHL3 did not show significant regulation by ELF5.

One-way ANOVA showed the effect of ELF5 on GRHL3 to be significant and 8 of 8 genes analysed showed a change in expression in response to induction of ELF5.

-The authors also state at line 322 that “ELF5 diminishes the transcriptional effects of estrogen”. That again is an overstatement. To be able to draw this conclusion, the authors should perform an experiment using ELF5NoDox and ELF5Dox treated with estrogen and then compare the results to be able to conclude this.

We have altered the abstract to read “RNA sequencing demonstrated that these changes altered estrogen-driven patterns of gene expression”, and at old line 322, now the final paragraph of the results, we now say “that induction of ELF5 alters patterns of estrogen-driven gene expression via transcriptional mechanisms,”. Throughout the new section entitled “ELF5 driven changes in gene expression.” We make it clear that we are comparing ELF5 action to established patterns of estrogen-driven gene expression.

Our experiments are conducted in estrogen-replete conditions, so this experiment has already been done. Induction of ELF5 has the same transcriptional effect as long term estrogen withdrawal (Fig 3A), and treatments that opposed the transcriptional effects of ELF5 included estrogen treatment (SFig9E). As we use estrogen replete conditions, the experiment has been done in the presence of ongoing estrogen stimulation, and these are the conditions required to detect an effect that opposes estrogen stimulation. These finding establish that ELF5 exerts an opposing transcriptional influence to estrogen on these genes and we see little to be gained from additional experiments adding estrogen to estrogen-replete conditions

-Also the title and main conclusions suggests reorganization of the ER cistrome, but how this occurs, which is the most interesting questions here, is not clear.

This is an important avenue for future investigation. Reporting that reorganisation of the ER cistrome occurs is the first step toward encouraging others in the field to consider ELF5 in their studies and this finding deserves to be reported promptly and prior to further investigation of the mechanism, which we regard as less important than the fact that that reorganisation of the ER cistrome occurs.

5. The Materials and Methods section falls short of how experiments were performed compromising the rigor and reproducibility of this study. For example, the authors should make clear how the dox treatment was done and what is being compared.

The methods and additional methods now provide a detailed description of the DOX treatment.

It’s difficult to find the information whether the comparison is done on MCF-7 cells or T47D, as well as dox treatment for 24 or 48h.

T47-D at 24 and 48 hours (stated in the figures), MCF-7 at 48 hours, stated in the methods). These times have been added to a new sentence in the the methods section.

What do the authors actually ChIP with? ELF5 itself or via the V5 tag?

Sentence added to the methods- ELF5 was IP with a mixture of anti V5 and anti ELF5 (SantaCruz N20) antibodies.

What does ELF no dox look like, was it Chip’d as a negative control? Was the data normalized to this? etc, etc.

There is no ELF5 no Dox sample as V5 is required for the ELF5 ChIP. Thus the ChIP results for ELF5 represent *de novo* ELF5-V5 binding to the genome induced by DOX.

Minor points:

1. Supp Fig 8 is impossible to read leaving the reader without a clear understanding of the data presented.

Figure 8 must be downloaded and read as a scalable .pdf, allowing the viewer to zoom in and out of regions of interest.

2. Some of the Supp data could be brought into the main figures. The reader is constantly having to move into the Supp figures to understand the main figures. For example, some of the ChEA analysis in Supp 9A could be moved into main figures.

To reduce the movement between main and supplementary we have reorganised the manuscript and added two new main figures. It is much improved as a result of this suggestion.

3. The Upset plots are sometimes very busy and difficult to follow.

Yes we also think upsets can be difficult, but no more so than multi set Venns. They are compact. We have used Venn’s as well where possible.

4. In line 220-221 the authors compared genes up-regulated or down-regulated upon estrogen deprivation to the ELF5 gene dataset. However, they do not specify where in Fig. 3A each of these datasets are and where the data for long-term estrogen deprivation is from.

We have named the molecular signature data base (MSigDB).

5. On line 255-256, MCMs are part of replication complexes not transcription complexes.

MCMs are members of the replication complex, this is their best-known function but MCMs are distributed far more widely on DNA than this function requires. As a helicase they also play a role in transcription (see **DOI:** 10.1128/MMBR.68.1.109-131.200) and this is the function to which we refer. Given this is a controversial area we have removed this sentence as it is not a key point.

6. On line 258-259, the authors cite 6 genes implicated in endocrine resistance that are differentially expressed by FOXA1 or ER1, however they are not at the list presented on Fig. 3D. Are these handful of genes enough for driving the phenotype?

Fig. 3D lists proteins known to physically interact with ER while the 6 genes listed at line 258 were identified by ChIP-seq and have not been reported to interact with ER. That 6 genes implicated in endocrine resistance are contained within the highly specific 26 gene candidate set suggests that others in this set have a similar effect. For example the effects of CD36 and CUEDC1 were published after our first literature scan of the 26 gene set.

**Reviewer #3:**

The authors provide a carefully done and interesting study with findings that very well support the role of the transcription factor ELF5 in playing a contributing role in endocrine resistance and breast cancer metastasis. ELF5 is known to have key roles in the development of the mammary gland and other tissues, and the authors now show that it also plays an important role in breast cancer in the acquisition of endocrine resistance and cancer progression to metastatic cancer. To investigate the mechanism by which ELF5 engenders endocrine resistance and metastasis, the authors examine genomic binding sites (ChIP-seq) of ELF5, ER, and FOXA1, gene expression by RNA-seq in breast cancer cells with dox-inducible elevated ELF5 expression, and use RIME and PLA to identify interacting proteins and to analyze and derive information and make correlations with RNA-seq and ChIP-seq publically available datasets from breast tumors displaying endocrine sensitivity or resistance, and metastases from endocrine-resistant patients. These analyses appear to be done rigorously and well and yield valuable new information on ELF5, ER, and FOXA1 cistromes and transcriptomes in MCF-7 and T47D cells with dox-inducible increased ELF5 expression and correlations with data from clinical breast cancers.

While other studies have shown that the ER cistrome can be modulated by other nuclear receptor transcription factors including progesterone receptor and retinoic acid receptor (RAR), this is the first study to examine the impact of elevated developmental transcription factor ELF5 on the ER- and FOXA1-regulated cistrome and transcriptome in breast cancer that underlie endocrine resistance and cancer progression. Elevated ELF5 changed the cistrome binding sites of ER and FOXA1, moving them to new genomic regions similar to those seen in breast cancers that have acquired resistance to endocrine therapy. RIME and PLA showed that ELF5 interacted with components of the ER transcriptional complex including members of the CoREST complex and DNA-PKcs, and that knockdown of DNA-PKcs altered ELF5-driven gene expression implicating their relevance in ELF5 activity. RNA-seq revealed that ELF5 also changed the expression of members of the ER transcription complex and of genes known previously to drive the acquisition of endocrine therapy resistance. Overall, the studies are well done and provide mechanistic insight on the role of ELF5 in altering estrogen action and engendering endocrine therapy resistance in cancer.

Questions
1. Why is the isoform 2 of ELF5 used in the cell lines in this study?

We carried out a detailed study of ELF5 isoform activity and showed that isoform 2 is the active isoform in breast cancer. We have added a reference to this paper (22). Isoform 2 is predominant.

2. Do the antibodies used detect all ELF5 isoforms, and are other isoforms present and of relevance in human tumors?

New SFig. 8 shows the isoform specificity of the N20 ELF5 antibody used as well as the antibody against the V5 tag.
